# Misrouting of v-ATPase subunit V0a1 dysregulates lysosomal acidification in a neurodegenerative lysosomal storage disease model

**DOI:** 10.1038/ncomms14612

**Published:** 2017-03-07

**Authors:** Maria B. Bagh, Shiyong Peng, Goutam Chandra, Zhongjian Zhang, Satya P. Singh, Nagarajan Pattabiraman, Aiyi Liu, Anil B. Mukherjee

**Affiliations:** 1 Section on Developmental Genetics, PEMG, Eunice Kennedy-Shriver National Institute of Child Health and Human Development, National Institutes of Health, Bethesda, Maryland 20892-1830, USA; 2 Laboratory of Molecular Immunology, National Institute of Allergy and Infectious Diseases, National Institutes of Health, Bethesda, Maryland 20892-1830, USA; 3 MolBox LLC, 8115 Fenton Street #304, Silver Spring, Maryland 20910, USA; 4 Biostatistics and Bioinformatics Branch, Division of Intramural Population Health Research, Eunice Kennedy-Shriver National Institute of Child Health and Human Development, National Institutes of Health, Bethesda, Maryland 20892-1830, USA

## Abstract

Defective lysosomal acidification contributes to virtually all lysosomal storage disorders (LSDs) and to common neurodegenerative diseases like Alzheimer's and Parkinson's. Despite its fundamental importance, the mechanism(s) underlying this defect remains unclear. The v-ATPase, a multisubunit protein complex composed of cytosolic V1-sector and lysosomal membrane-anchored V0-sector, regulates lysosomal acidification. Mutations in the *CLN1* gene, encoding PPT1, cause a devastating neurodegenerative LSD, INCL. Here we report that in *Cln1*^*−/−*^ mice, which mimic INCL, reduced v-ATPase activity correlates with elevated lysosomal pH. Moreover, v-ATPase subunit a1 of the V0 sector (V0a1) requires palmitoylation for interacting with adaptor protein-2 (AP-2) and AP-3, respectively, for trafficking to the lysosomal membrane. Notably, treatment of *Cln1*^*−/−*^ mice with a thioesterase (Ppt1)-mimetic, NtBuHA, ameliorated this defect. Our findings reveal an unanticipated role of *Cln1* in regulating lysosomal targeting of V0a1 and suggest that varying factors adversely affecting v-ATPase function dysregulate lysosomal acidification in other LSDs and common neurodegenerative diseases.

## Abstract (web summary)

Lysosomal acidification defects have been implicated in various neurodegenerative disorders. Bagh *et al*. show that the V0a1 subunit of v-ATPase requires palmitoylation for correct sorting and trafficking to the lysosome membrane, and that such a process is impaired in a mouse model of a devastating neurodegenerative lysosomal storage disease, INCL.

In eukaryotic organisms, the lysosome is the primary organelle for intracellular digestion^1^. It contains >50 hydrolases that require acidic pH for optimal degradative function^2^. Thus, lysosomal acidification is of fundamental importance in the degradation of macromolecules of intra- and extracellular origin, which are delivered to the lysosome. Moreover, it has been reported that dysregulation of lysosomal acidification contributes to pathogenesis in virtually all lysosomal storage disorders (LSDs)^3^,^4^ including several neuronal ceroid lipofuscinoses (NCLs)^5^, commonly known as Batten disease. Furthermore, defective regulation of lysosomal pH has also been reported in common neurodegenerative diseases like Alzheimer's^6^,^7^ and Parkinson's^8^. However, despite intense studies, the mechanism(s) underlying defective lysosomal acidification in these diseases remains largely unclear.

Lysosomal acidification is regulated by vacuolar ATPase (v-ATPase)^9^,^10^,^11^, a multisubunit protein complex composed of the cytosolic V1 sector and the lysosomal membrane-anchored V0-sector. Reversible assembly of V1/V0 sectors on lysosomal membrane maintains functionally active v-ATPase, the proton pump of the cell, which regulates lysosomal acidification^9^. The NCLs represent a group of the most prevalent neurodegenerative LSDs caused by mutations in >13 different genes (called the *CLNs*)^12^. The infantile NCL (INCL)^13^, a devastating neurodegenerative LSD, is caused by inactivating mutations in the *CLN1* gene^14^, which encodes palmitoyl-protein thioesterase-1 (PPT1)^15^.

Palmitoylation (also called S-acylation)^16^,^17^ is a reversible post-translational modification in which a long-chain fatty acid (predominantly palmitate) is attached to specific cysteine residues in polypeptides via thioester linkage. In the mammalian genome, 23 genes encode palmitoyl-acyl-transferases (PATs), which are evolutionarily conserved, cysteine-rich proteins containing Asp-His-His-Cys (DHHC) domains that catalyse palmitoylation^17^. In contrast, there are four thioesterases that have been characterized, two of which are cytosolic (for example, acyl-protein thioesterase-1 (Apt1) and Apt2) and two (for example, PPT1 and PPT2) are localized to the lysosome. Dynamic palmitoylation (palmitoylation–depalmitoylation)^17^,^18^,^19^ requiring coordinated action of both the DHHC-PATs and PPTs maintains steady-state membrane localization and function of numerous important proteins^17^,^18^,^19^,^20^, especially in the brain^21^. By catalysing depalmitoylation, thioesterases also facilitate recycling or degradation of proteins that undergo palmitoylation^22^.

In this study, we tested a hypothesis that one or more subunits of v-ATPase requires S-palmitoylation for endosomal sorting, trafficking and reversible assembly of V1/V0 on lysosomal membrane, which is essential for regulating lysosomal pH and that Ppt1 deficiency disrupts v-ATPase activity impairing its proton transport function, thereby dysregulating acidification of lysosomal lumen. Our results show that lysosomal membrane-anchored V0 sector isoform a1 (V0a1) subunit of v-ATPase undergoes S-palmitoylation, which is required for its sorting and trafficking to the lysosomal membrane. This process appears to be defective in Ppt1-deficient *Cln1*^*−/−*^ mice. Notably, we demonstrate that treatment of these mice with a thioesterase (Ppt1)-mimetic small molecule, N-tert (Butyl) hydroxylamine (NtBuHA), restores near-normal v-ATPase activity and rescues the defective lysosomal acidification phenotype.

## Results

### Lysosomal pH and v-ATPase activity in *Cln1*^*−/−*^ mice

**Figure 1:**
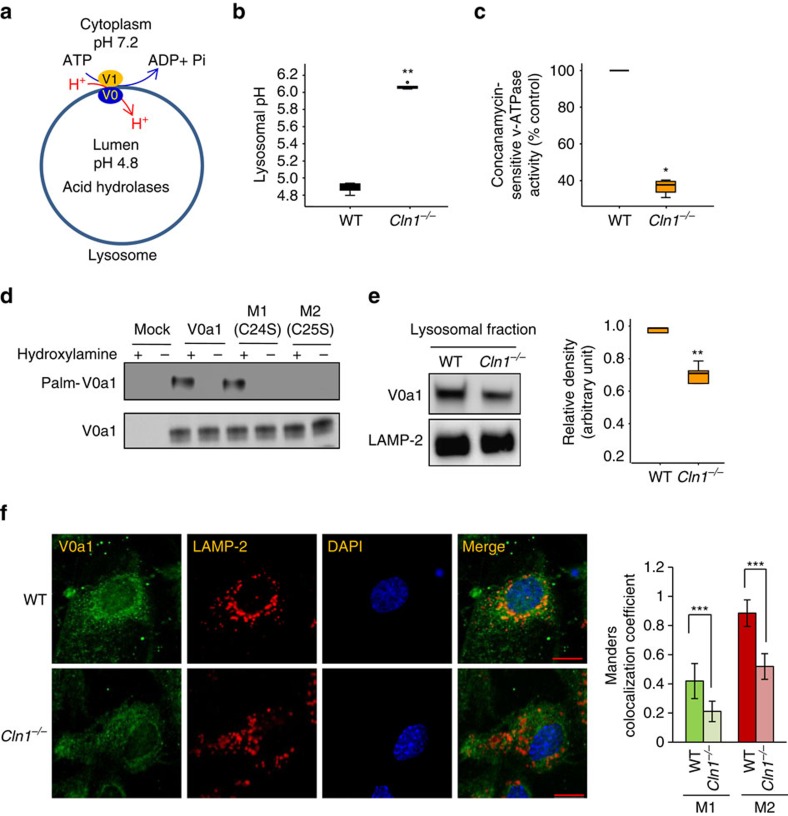
Palmitoylation of V0a1 of v-ATPase and regulation of lysosomal acidification. (**a**) Schematic diagram showing the localization of the cytosolic V1 sector (yellow) and V0 sector (blue) of v-ATPase on lysosomal membrane. The V1 sector hydrolyses ATP to ADP generating energy for the lysosomal membrane-anchored V0 sector to translocate protons from the cytosol to the lysosomal lumen for acidification. (**b**) Lysosomal pH in WT and *Cln1*^*−/−*^ neurons. Lysosomal pH was measured using Oregon green dextran and TMR dextran as described in the Methods. Note that compared with WT neurons the lysosomal pH in *Cln1*^*−/−*^ cells is significantly elevated (WT: ∼4.88±0.05 versus *Cln1*^*−/−*^: ∼5.96±0.02; *n*=6, **P<0.01). (**c**) Enzymatic activity of v-ATPase in purified lysosomal fractions from brain tissues of WT and *Cln1*^*−/−*^ mice (*n*=4), *P<0.05. (**d**) Western blot analysis of total lysates of HEK-293 cells transfected with the empty vector (mock control), V0a1, mutant (Cys24Ser) V0a1 or mutant (Cys25Ser) V0a1 constructs. ABE assay was used to confirm that Cys-25 but not Cys-24 in V0a1 undergoes palmitoylation in V0a1. (**e**) Levels of V0a1 in lysosomal fractions isolated from the brain tissues of WT and *Cln1*^*−/−*^ mice (*n*=4, **P<0.01. (**f**) Confocal imaging of primary neurons from WT and *Cln1*^*−/−*^ mice showing colocalization of V0a1 with Lamp2. Colocalization between V0a1 and Lamp2 was assessed using the Manders' colocalization coefficients M1 (green) and M2 (red), which, respectively, represent the overlap with green pixels as the denominator and vice versa (*n*=23 for WT and *n*=27 for *Cln1*^*−/−*^, ***P<0.001), scale bars, 5 μm.

The v-ATPase is the proton pump localized to the late endosomal/lysosomal membrane^9^ that regulates the acidic pH in the lysosomal lumen (Fig. 1a). The V1 sector of v-ATPase hydrolyses ATP to generate energy for the lysosomal membrane-anchored V0-sector, which translocates protons from the cytoplasm to the lysosomal lumen for acidification^9^,^10^,^11^. Since in our study we used *Cln1*^*−/−*^ mice^23^, which mimic INCL, as the model for neurodegenerative LSDs, we first determined whether lysosomal acidification is dysregulated in these animals. Accordingly, we loaded the cultured neurons from wild type (WT) and *Cln1*^*−/−*^ mice with a fluorescent dye, lysosensor DND-189. The fluorescence intensity of this dye is inversely correlated with the pH in lysosomes. We found that compared with the neurons from WT mice (Supplementary Fig. 1a) those of their *Cln1*^*−/−*^ littermates, which mimic INCL, had markedly reduced fluorescence intensity (Supplementary Fig. 1b) indicating elevated lysosomal pH. We confirmed these results by using a fluorometric assay^24^ to generate a standard curve (Supplementary Fig. 1c), which was used to quantitate the lysosomal pH in WT and *Cln1*^*−/−*^ neurons. We found that compared to WT littermates the neurons from *Cln1*^*−/−*^ mice had a significantly higher lysosomal pH (WT:∼4.88±0.05 versus *Cln1*^*−/−*^: ∼5.96±0.02; *P*<0.01; Fig. 1b). Notably, the v-ATPase activity in lysosomal fractions from brain tissues of *Cln1*^*−/−*^ mice was strikingly lower compared with that of the WT mice (Fig. 1c). Taken together, these results confirmed that in our LSD model lysosomal acidification is dysregulated most likely due to low v-ATPase activity.

### Cysteine-25 in v-ATPase subunit V0a1 requires palmitoylation

The V0 sector of v-ATPase is anchored to the lysosomal membrane, and reversible assembly of V1/V0 regulates the v-ATPase activity^9^,^10^,^11^. The V0 sector consists of six different subunits (a, c, c″, d, e and Ac 45 in mammals)^9^. The a-subunit has four isoforms (a1–a4), which are expressed in an organ- and cell type-specific manner^10^. For example, the V0a1 is expressed at a high level in the brain, the a2 isoform in the liver as well as in the kidneys, while the a3 isoform is expressed in osteoclasts^25^. Notably, in the yeast the V0a1 plays a pivotal role in the reversible V1/V0 assembly^26^ essential for maintaining v-ATPase activity^9^. Since many important proteins in the brain require the reversible S-palmitoylation for steady-state membrane localization, protein–protein interaction and function^16^,^17^,^18^,^19^, we reasoned that one or more subunits of the V0 sector may be S-palmitoylated to regulate reversible V1/V0 assembly on lysosomal membrane.

To determine whether any of the V0 sector subunits are palmitoylated, we first analysed the peptide sequences of all the subunits in this sector by CSS-Palm^27^, a computer programme that predicts the cysteine residues in polypeptides that are likely to undergo S-palmitoylation. The results of this analysis predicted that Cys-25 in V0a1, Cys-11 in V0a2 and V0a4 are the likely candidate sites for palmitoylation (Supplementary Fig. 1d). Notably, only the V0a1 isoform is preferentially expressed at a high level in the brain^20^,^21^, while V0a2 is expressed at a very low level in this organ. Although a low-level expression of V0a3 has been reported in the brain^28^, CSS-palm analysis predicted no palmitoylation sites. The expression of the other isoforms is not readily detectable in the brain. Accordingly, we sought to determine whether V0a1 and V0a2 are palmitoylated using acyl-biotinyl exchange (ABE) method^29^. We found that, while V0a1 was palmitoylated (Fig. 1d), as predicted, no palmitoylation signal was detectable in V0a2 (Supplementary Fig. 1e). To further confirm these results, we transfected HEK-293 cells with V0a1–cDNA constructs in which either Cys-24 or Cys-25 was mutated to Ser. The results showed that only Cys25Ser mutation abrogated palmitoylation of V0a1 (Fig. 1d). Taken together, these results confirmed that Cys-25 in V0a1 is the only palmitoylated residue, which is also evolutionarily conserved across phyla (Supplementary Fig. 1f).

### Lysosomal V0a1 localization is disrupted in *Cln1*^*−/−*^ mice

To delineate the cause(s) of the strikingly low v-ATPase activity in the brain lysosomal fractions from *Cln1*^*−/−*^ mice, we first determined V0a1 mRNA levels to rule out the possibility that it is not transcriptionally downregulated. The results showed no significant difference in V0a1-mRNA levels between WT and *Cln1*^*−/−*^ mice (Supplementary Fig. 2a). We also measured the V0a1-protein levels in total brain lysates and in purified lysosomal fractions from the brain tissues of WT and *Cln1*^*−/−*^ mice. We found that, while V0a1-protein levels in total brain lysates from WT and *Cln1*^*−/−*^ mice were virtually identical (Supplementary Fig. 2b), those in the lysosomal fractions from *Cln1*^*−/−*^ mouse brain were significantly lower (Fig. 1e). These results were further confirmed by confocal imaging of cultured neurons, which showed that in *Cln1*^*−/−*^ cells a substantially low level of V0a1 immunofluorescence colocalized with the lysosomal membrane marker, Lamp 2 (Fig. 1f). Cumulatively, our results strongly suggested that in *Cln1*^*−/−*^ mice the localization of V0a1 to the lysosomal membrane was disrupted.

### V0a1 palmitoylation facilitates its endosomal trafficking

Lysosomal membrane proteins are transported along the biosynthetic pathway consisting of the endoplasmic reticulum (ER), the Golgi apparatus, the trans-Golgi network, the plasma membrane and the endosome^30^. These proteins contain the tyrosine- and di-leucine-based endocytic sorting signals that are recognized by the adaptor proteins (APs) localized within the clathrin-coated vesicles. Four AP complexes (that is, AP-1, AP-2, AP-3 and AP-4) interact with the cargo proteins for sorting and trafficking to the lysosomal membrane. The sorting of the lysosomal membrane proteins from the ER–Golgi to the endosomal/lysosomal compartment may occur either directly, or they are first transported to the plasma membrane followed by endocytosis to the early endosome by AP-2 and from there via AP-3 to the late endosomal/lysosomal membrane^30^. Notably, it has been reported that the clathrin adaptors associate with the V0a subunit of v-ATPase^31^,^32^,^33^.

**Figure 2:**
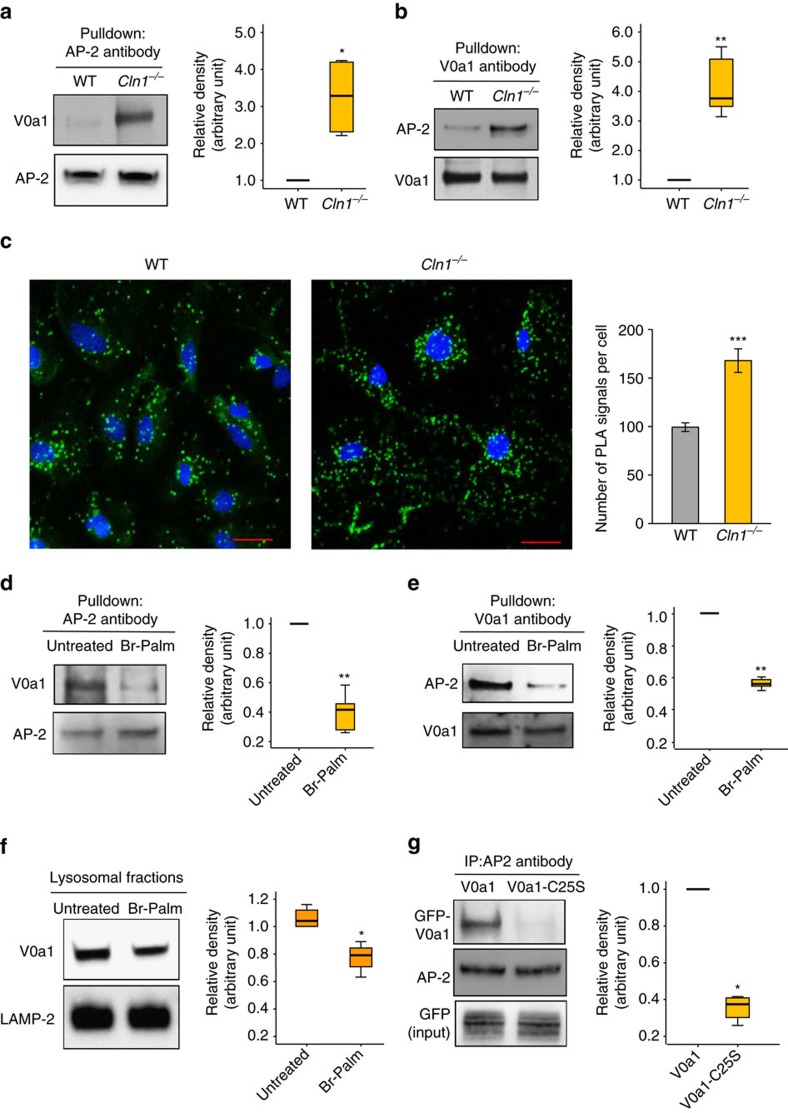
Palmitoylation of V0a1 promotes its interaction with AP-2. (**a**) Antibody to AP-2 pulls down V0a1 in total brain lysates from WT and *Cln1*^*−/−*^ mice (*n*=4, *P<0.05). (**b**) Antibody to V0a1 pulls down AP-2 in total brain lysates from WT and *Cln1*^*−/−*^ mice (*n*=4, **P<0.01). (**c**) Confocal imaging of cultured neurons isolated from WT and *Cln1*^*−/−*^mouse brains were performed to determine interaction of V0a1 with AP-2 by PLA reaction (*n*=129 for WT and *n*=62 for *Cln1*^*−/−*^, ***P<0.001). (**d**,**e**) Pull-down experiments using either AP-2- or V0a1 antibody were performed to detect V0a1 and AP-2, respectively, in total lysates from untreated and bromopalmitate-treated (Br-palm) WT brain slices (*n*=5, **P<0.01). (**f**) Western blot analysis and quantitation of V0a1 in isolated lysosomal fractions from untreated and bromopalmitate (Br-palm)-treated WT brain slices (*n*=4, *P<0.05). (**g**) HEK-293 cells were transfected with WT GFP-V0a1 and mutant (Cys25Ser) GFP-V0a1-construct and pull-down experiments were performed using AP-2 antibody to detect GFP-V0a1 (*n*=4), *P<0.05, scale bars, 20 μm.

Since V0a1 is localized to the lysosomal membrane, we analysed its peptide sequence for tyrosine- and dileucine-based endocytic sorting signals recognized by the APs. We found that both of the tyrosine and dileucine motifs are represented in V0a1, and are evolutionarily conserved across phyla (Supplementary Fig. 1f). Accordingly, we performed pull-down assays using antibodies to various APs or V0a1 to determine whether there was any difference in V0a1 interaction with the APs between WT and *Cln1*^*−/−*^ mice. We found that there was no difference in the level of V0a1–AP-1 interaction in brain tissues from WT and *Cln1*^*−/−*^ mice (Supplementary Fig. 3a,b). However, the pull-down assay using AP-2- or V0a1 antibody showed a substantially higher level of V0a1–AP-2 interaction in *Cln1*^*−/−*^ mouse brain (Fig. 2a,b). Consistent with these results, confocal imaging using proximity ligation assay also showed a markedly higher level of V0a1–AP-2 interaction in *Cln1*^*−/−*^ mouse neurons (Fig. 2c). To further confirm this finding, we performed short hairpin RNA (shRNA) knockdown of AP-2 in HEK-293 cells. We found that AP-2 knockdown (Supplementary Fig. 3c) caused substantially increased levels of V0a1 in the plasma membrane fractions (Supplementary Fig. 3d) compared with those in mock shRNA-transfected HEK-293 cells. To determine whether AP-2 interacts with either tyrosine or dileucine motif for its V0a1 sorting, we transfected HEK-293 cell constructs containing either WT or mutants in the tyrosine or dileucine motifs of V0a1 and performed pull-down assays using AP-2 antibody. The results showed that mutation in both tyrosine and dileucine motifs adversely affected the interaction of V0a1 with AP-2 (Supplementary Fig. 3e). These results strongly suggested that AP-2 plays a critical role in endocytic trafficking of V0a1.

### Palmitoylation of V0a1 regulates its interaction with AP-2

Since palmitoylation enhances protein–protein interactions^16^,^17^,^18^,^19^, we reasoned that V0a1 palmitoylation may enhance its interaction with AP-2, facilitating endocytic sorting and trafficking to the early endosome. We also reasoned that in the early endosome dynamic S-palmitoylation (palmitoylation–depalmitoylation) of V0a1 may be required for its dissociation from AP-2 in order to interact with AP-3 essential for transport to the late endosomal/lysosomal membrane. Accordingly, we incubated thin (0.5 mm) brain slices from WT mice in tissue culture media containing 50 μM bromopalmitate (an inhibitor of palmitoylation) for 12 h, and performed pull-down assays using either V0a1- or AP-2 antibody. The results showed that V0a1–AP-2 interaction in bromopalmitate-treated brain slices was substantially lower compared with that in the untreated controls (Fig. 2d,e). Moreover, western blot analyses showed that, while V0a1 levels in total lysates of untreated and bromopalmitate-treated brain slices were virtually identical (Supplementary Fig. 4a), those in the purified lysosomal fractions from bromopalmitate-treated slices were significantly lower (Fig. 2f). Furthermore, confocal imaging of cultured WT neurons also confirmed that the bromopalmitate treatment markedly reduced colocalization of V0a1 with the lysosomal membrane marker, Lamp 2 (Supplementary Fig. 4b). While bromopalmitate suppresses palmitoylation by inhibiting PATs, it may also inhibit other enzymes, unrelated to the PATs^34^. Accordingly, we sought to further confirm the results of bromopalmitate experiments by transfecting HEK-293 cells with either a green fluorescent protein (GFP)-tagged V0a1–cDNA construct or the V0a1-mutant (Cys25Ser)–cDNA construct and performed pull-down assays using AP-2 antibody. The results showed that Cys-25 palmitoylation is essential for V0a1–AP-2 interaction as the V0a1-mutant (Cys25Ser) had significantly reduced interaction with AP-2 (Fig. 2g). To further confirm the role of S-palmitoylation on lysosomal localization of V0a1, we purified the lysosomal fractions from HEK-293 cells transfected with either WT V0a1–cDNA construct or the V0a1-mutant (Cys25Ser)–cDNA construct. While the western blot analyses of total lysates of the cells transfected with WT V0a1 and mutant V0a1 (Cys25Ser) constructs showed virtually identical levels of V0a1-protein (Supplementary Fig. 4c), the lysosomal fractions showed significantly reduced levels of mutant V0a1 (Supplementary Fig. 4d). Cumulatively, these results suggested that V0a1 palmitoylation is critical for its interaction with AP-2, which is essential for its endocytic sorting and trafficking.

### Ppt1 deficiency impairs V0a1 trafficking to the lysosome

**Figure 3:**
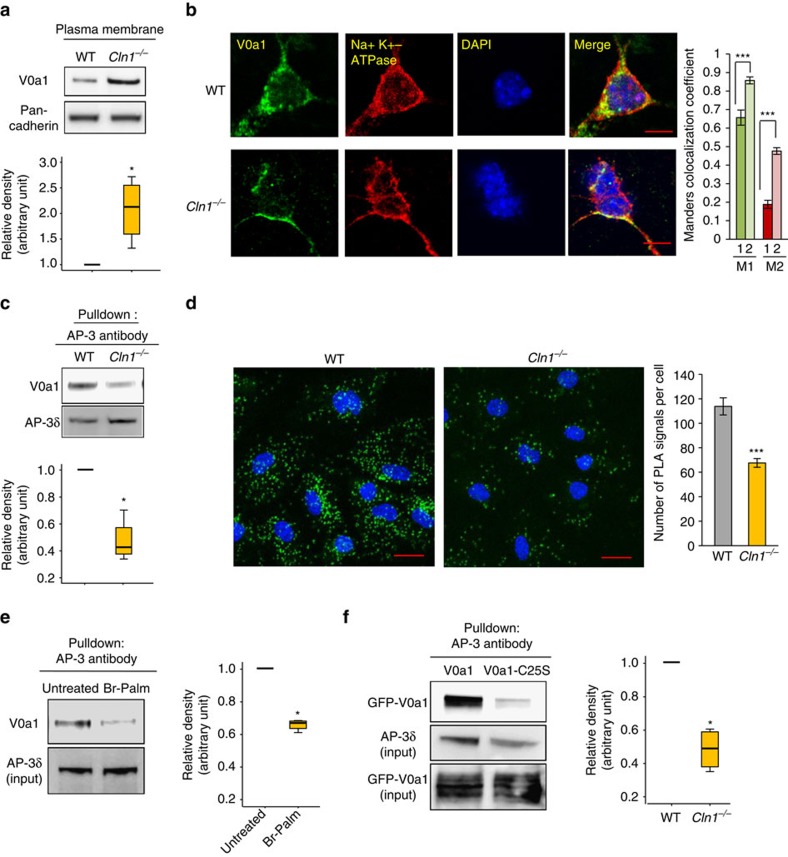
In *Cln1*^*−/−*^ mice V0a1 is misrouted to plasma membrane preventing its interaction with AP-3. (**a**) Western blot analysis and densitometric quantitation of V0a1 in isolated plasma membrane fraction from WT and *Cln1*^*−/−*^ mouse brain (*n*=4, *P<0.05). (**b**) Localization of V0a1 in the plasma membrane in WT and *Cln1*^*−/−*^ neurons using Na^+^, K^+^-ATPase as membrane marker. Colocalization between V0a1 and Na^+^, K^+^-ATPase was assessed using the Manders' colocalization coefficients M1 and M2 (*n*=18 for WT and *n*=22 for *Cln1*^*−/−*^, ***P<0.001; scale bars, 5 μm. (**c**) Pull-down assay with AP-3 antibody detects V0a1 in total brain lysates from WT and *Cln1*^*−/−*^ mouse brain (*n*=4, *P<0.05). (**d**) Confocal imaging of PLA reaction showing V0a1 and AP-3δ interaction in neurons isolated from WT and *Cln1*^*−/−*^mouse brain (*n*=188 for WT and *n*=158 for *Cln1*^*−/−*^, ***P<0.001); scale bars, 20 μm. (**e**) Pull-down assay with AP-3 antibody using total lysates from untreated (lane 1) and bromopalmitate-treated (lane 2) WT brain slices to detect V0a1 and its densitometric quantitation (*n*=4, *P<0.05). (**f**) HEK-293 cells were transfected with WT GFP-V0a1 and GFP-V0a1-Cys25Ser mutant construct, and pull-down experiments were conducted with AP-3 antibody to detect GFP-V0a1,*P<0.05(*n*=4).

To determine whether Ppt1 deficiency in *Cln1*^*−/−*^ mice disrupted the clathrin–AP-2-mediated endocytosis of V0a1 from the plasma membrane, we performed western blot analyses of plasma membrane fractions from WT and *Cln1*^*−/−*^ mouse brains using V0a1 antibody. We found that, compared with the WT controls, significantly higher levels of V0a1 were associated with the plasma membrane fractions from *Cln1*^*−/−*^ mouse brain (Fig. 3a). Confocal imaging studies confirmed that in *Cln1*^*−/−*^ mice high levels of V0a1 immunofluorescence colocalized with that of the plasma membrane marker, Na^+^/K^+^ ATPase (Fig. 3b). These results suggested that Ppt1 deficiency in *Cln1*^*−/−*^ mice may have impaired S-palmitoylation of V0a1, and disrupted its endocytic sorting and trafficking.

Following arrival at the early endosome, the transport of proteins to the late endosomal/lysosomal membrane is mediated by AP-3 (ref. 30). This is supported by the fact that mutations in the β3A subunit of AP-3 disrupt endosomal trafficking of proteins to the lysosomal membrane causing an LSD-like disease called Hermansky-Pudlak syndrome^35^. Therefore, we sought to determine whether in *Cln1*^*−/−*^ mice impaired dissociation of V0a1 from AP-2 is essential for its interaction with AP-3. Accordingly, we performed pull-down assays using antibodies to AP-3δ. The results showed that a substantially reduced level of V0a1 was pulled down from *Cln1*^*−/−*^ mouse brain lysates compared with those of the WT controls (Fig. 3c). Confocal microscopy using antibodies to V0a1 and AP-3δ, respectively, in proximity ligation assay showed that there was a substantially reduced level of AP-3–V0a1 interaction in cultured neurons from *Cln1*^*−/−*^ mice (Fig. 3d). To assess whether V0a1 palmitoylation is critical for its interaction with AP-3, we treated brain slices from WT mice with bromopalmitate and performed pull-down assays using AP-3 antibody. We found that in bromopalmitate-treated brain slices from WT mice V0a1–AP-3 interaction was substantially reduced (Fig. 3e). Furthermore, pull-down assays using HEK-293 cells transfected with the palmitoylation site-mutant V0a1 (Cys25Ser)–cDNA construct also showed a marked reduction in V0a1–AP-3 interaction (Fig. 3f). Taken together, these results strongly suggested that palmitoylation of V0a1 is essential for its trafficking from the early endosome to the late endosomal/lysosomal membrane.

### Ppt1 and V0a1 localize to endosomal/lysosomal compartments

**Figure 4:**
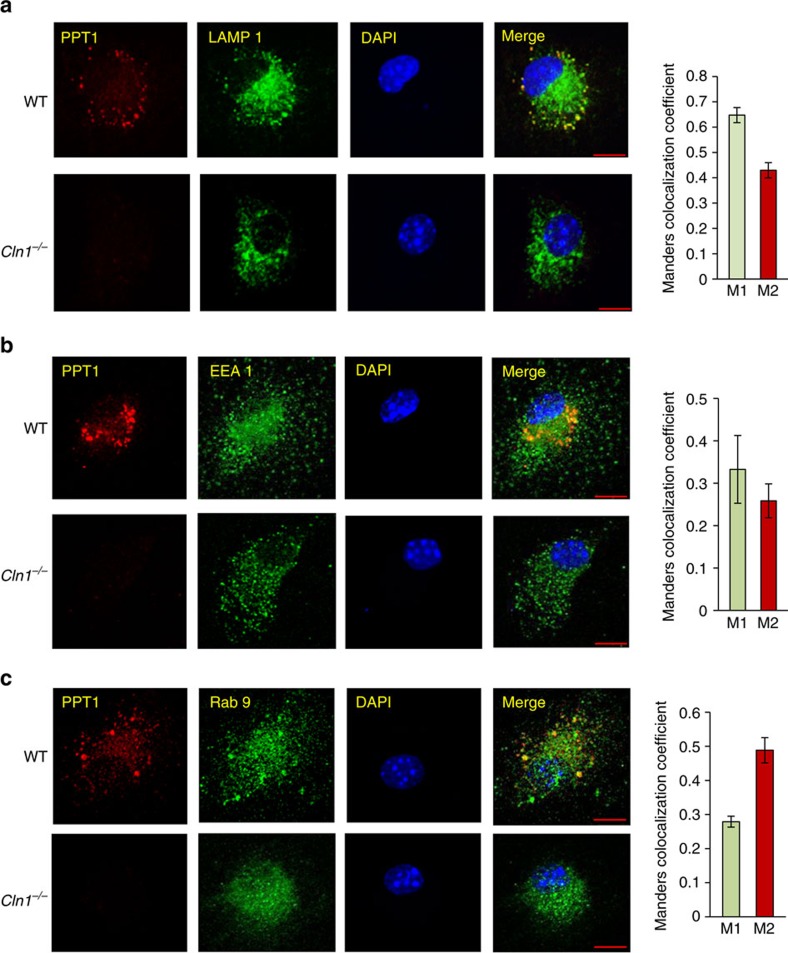
Endosomal and lysosomal localization of Ppt1. (**a**) Colocalization of Ppt1 with lysosome marker (LAMP 1) in WT and *Cln1*^*−/−*^ brain cells (*n*=34). (**b**) Colocalization of Ppt1 with early endosome marker (EEA1) in WT and *Cln1*^*−/−*^ cells (*n*=6). (**c**) Colocalization of Ppt1 with late endosome marker (Rab 9) in WT and *Cln1*^*−/−*^ cells (*n*=5). Colocalization of Ppt1 with the endosomal markers in WT cells was assessed using the Manders' colocalization coefficients M1 and M2. Scale bars, 5 μm.

**Figure 5:**
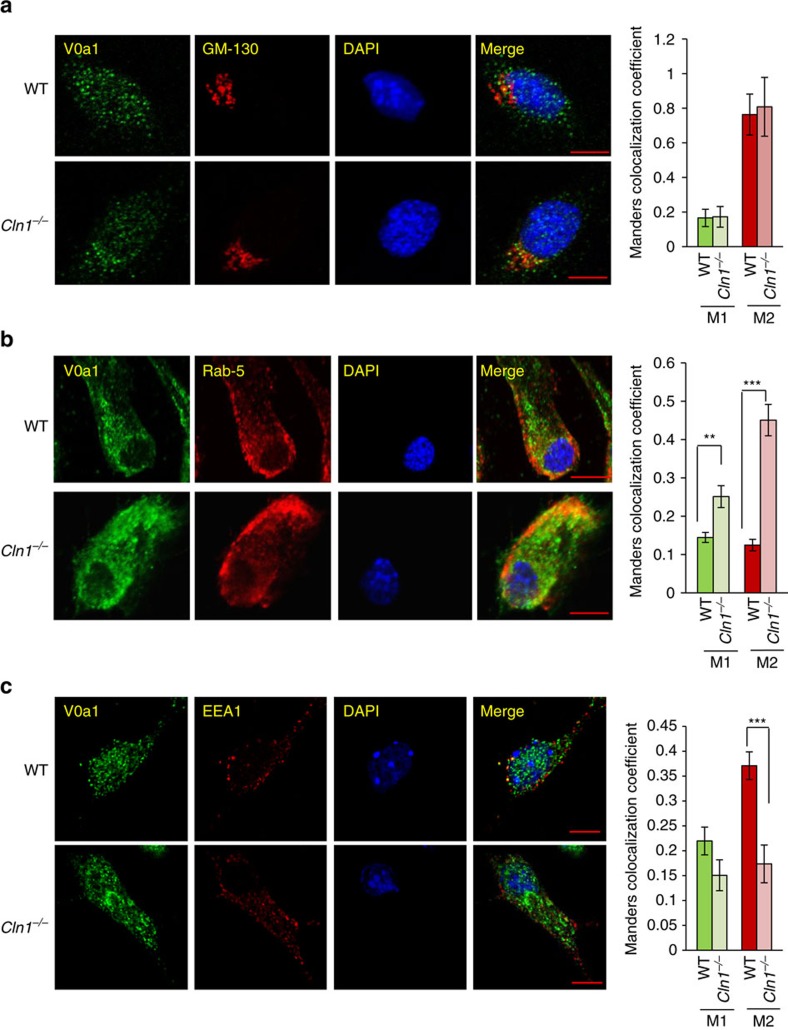
Localization of V0a1 in the Golgi and in early endosome. (**a**) Confocal imaging colocalizing the Golgi marker (GM-130) with V0a1 in cortical neurons from WT and *Cln1*^*−/−*^ mouse brain (*n*=26 for WT and 20 for *Cln1*^*−/−*^). (**b**) Colocalization of V0a1 with early sorting endosome (Rab-5) using confocal imaging of WT and *Cln1*^*−/−*^ neurons (*n*=31 for WT and 27 for *Cln1*^*−/−*^, **P<0.01, ***P<0.001). (**c**) Colocalization of V0a1 with early endosome marker (EEA1) in WT and *Cln1*^*−/−*^ neuronal cells (*n*=12 for WT and 10 for *Cln1*^*−/−*^, ***P<0.001). Scale bars, 5 μm.

**Figure 6:**
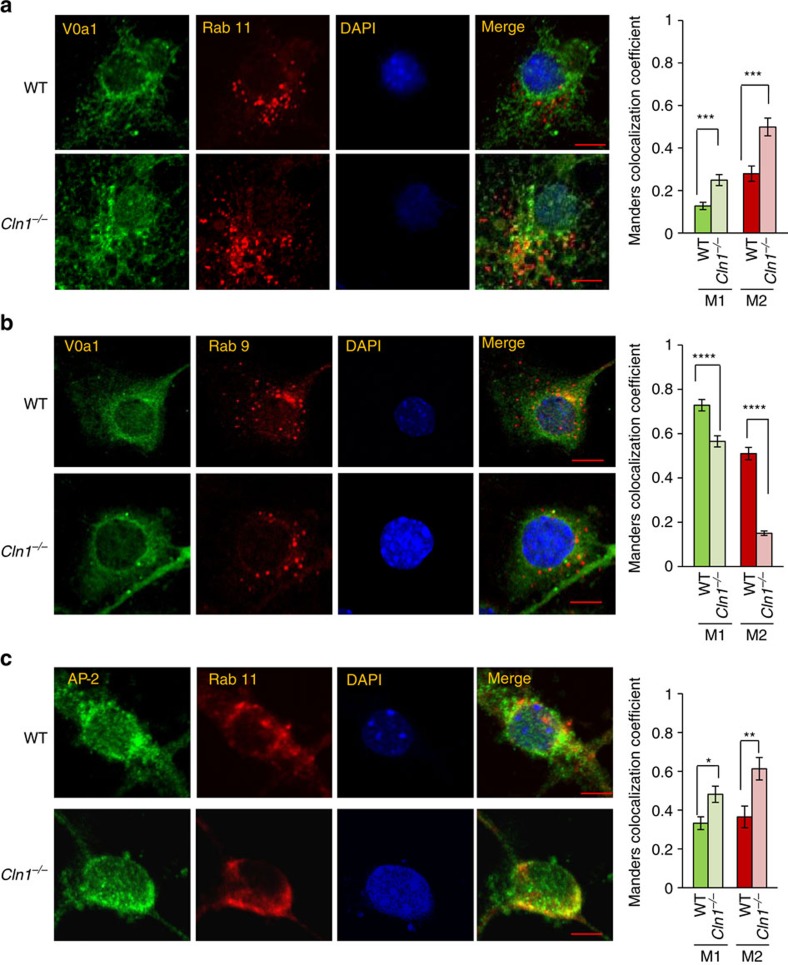
Endosomal localization of V0a1 and AP-2. (**a**) Colocalization of V0a1 with Rab11 (Recycling endosome marker) using confocal imaging of WT and *Cln1*^*−/−*^ neurons (*n*=27 for WT and 24 for *Cln1*^*−/−,*^ ***P<0.001). (**b**) Colocalization of V0a1 with late endosome marker (Rab 9) in isolated neurons from WT and *Cln1*^*−/−*^mouse brain (*n*=41 for WT and 31 for *Cln1*^*−/−*^, ****P<0.0001). (**c**) Colocalization of AP-2 with Rab11 in WT and *Cln1*^*−/−*^ neurons (*n*=14 for WT and *n*=19 for *Cln1*^*−/−*^). *P<0.05, **P<0.01. Scale bars, 5 μm.

To determine whether Ppt1 deficiency in *Cln1*^*−/−*^ mice disrupted endocytic sorting and trafficking of V0a1, we first sought to delineate whether in WT mice Ppt1 is present in endosomal/lysosomal compartments. Accordingly, we performed confocal imaging to colocalize Ppt1 immunoreactivity with that of the endosomal/lysosomal markers in cultured neurons from WT and *Cln1*^*−/−*^ mice. Our results showed that in WT neurons Ppt1 not only colocalized with the lysosomal marker, Lamp1 (Fig. 4a), but also with the early endosomal marker, EEA1 (Fig. 4b), and the late endosomal marker, Rab 9 (Fig. 4c). Interestingly, the colocalization of V0a1 with the ER marker, calnexin (Supplementary Fig. 5a), and the Golgi marker, GM-130 (Fig. 5a), in WT and *Cln1*^*−/−*^ neurons were virtually identical. Notably, in *Cln1*^*−/−*^ neurons colocalization of V0a1 with that of the sorting early endosomal marker, Rab-5 (Fig. 5b) was significantly higher compared to WT cells, while that with EEA1 early endosomal marker (Manders coefficient-M2) was substantially lower compared with that of the WT controls (Fig. 5c). Unexpectedly, in neurons from *Cln1*^*−/−*^ mice a markedly higher level of V0a1 colocalized with the recycling endosomal marker, Rab11 (Fig. 6a). Moreover, colocalization of V0a1 with the late endosomal marker, Rab 9, was significantly reduced in neurons from *Cln1*^*−/−*^ mice (Fig. 6b). Recent reports indicate that coat proteins may remain on the vesicles post budding and may even help the vesicle to fuse with the target membrane^36^. These studies indicate that some portions of the coat may still be there on the vesicles till the tethering steps. Although most of the studies have been done with COP1- and COP11-mediated transport, the reports indicate that the process of uncoating takes place in several phases. It has been reported that AP-2 interacts with Scrib1 to control N-methyl-D-aspartate receptor (NMDAR) post-endocytic traffic^37^. Accordingly, we performed colocalization studies with AP-2 and Rab11 in neurons isolated from WT and *Cln1*^*−/−*^ mouse brain. The results indicate that there is colocalization of AP-2 with Rab11 under the present experimental conditions, and the colocalization is significantly increased in the *Cln1*^*−/−*^ cells under similar conditions (Fig. 6c). This result suggests that the increased interaction of V0a1 with AP-2 promotes recycling of V0a1 through the Rab-5- and Rab11-positive vesicles.

Since V0a1 in *Cln1*^*−/−*^ mice was misrouted to the plasma membrane, we determined the levels of V1 sector subunit A1 (V1A1) in lysosomal fractions from WT and *Cln1*^*−/−*^ mouse brains. We found that V1A1–protein levels were significantly lower in lysosomal fractions from *Cln1*^*−/−*^ mouse brain compared with those of the WT controls (Supplementary Fig. 5b).Taken together, these results strongly suggested that in *Cln1*^*−/−*^ mice misrouting of V0a1 to the plasma membrane may have reduced V1A1/V0a1 assembly on lysosomal membrane, which disrupted v-ATPase function.

### V0a1–S-palmitoylation enables its endosomal/lysosomal sorting

**Figure 7:**
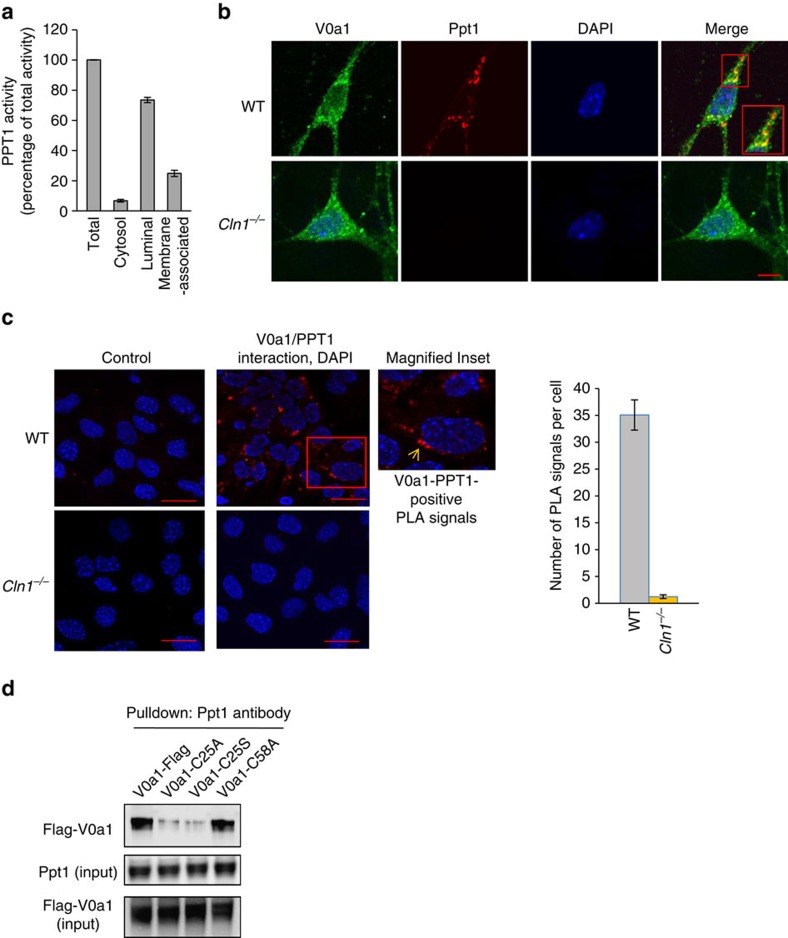
S-acylated V0a1 is a potential substrate of Ppt1. (**a**) Ppt1 activity in various subcellular fractions (*n*=4). (**b**) Confocal imaging of cultured neurons from WT and *Cln1*^*−/−*^mice showing colocalization of Ppt1 immunoreactivity with that of V0a1 in WT neurons only; scale bar, 5 μm. (**c**) Proximity ligation assay with mouse-V0a1 and rabbit-Ppt1 antibody to show possible interaction between V0a1 and Ppt1. The red dots are the positive signals showing V0a1–Ppt1 interaction in WT cells. Cells from *Cln1*^*−/−*^mice lacking Ppt1 and WT cells with no primary antibody served as the controls (*n*=252 for WT and *n*=102 for *Cln1*^*−/−*^). Scale bars, 20 μm. (**d**) Pull-down experiment with Ppt1 antibody using lysates of HEK-293 cells transfected with FLAG-tagged WT-V0a1, C25A-, C25S- and C58A-mutant constructs.

Our results indicate that V0a1 requires palmitoylation for its interaction with AP-2 and AP-3 respectively, facilitating its endosomal sorting and transport to the lysosomal membrane. It may also require depalmitoylation followed by repalmitoylation in order to dissociate from AP-2 and associate with AP-3. This raises a question as to how PPT1, generally assumed to be a thioesterase in the lysosomal lumen, may catalyse depalmitoylation of V0a1 localized to the cytosolic face of the lysosomal membrane. However, while PPT1 has been reported to be a lysosomal enzyme^38^,^39^, its extralysosomal localization in the central nervous system has also been demonstrated^40^,^41^. In addition, cell fractionation studies using mouse primary neurons and brain tissues as well as cryoimmunoelectron microscopy have shown that PPT1 localizes to neuronal synapses^42^. Moreover, the immunoelectron microscopic localization of PPT1 on synaptic vesicles (SVs) at the nerve terminals in WT mouse brain has been suggested to facilitate SV recycling to maintain SV-pool size in the synapses^43^ as the lack of Ppt1 has been reported to cause progressive decline in SV-pool size in cultured neurons from *Cln1*^*−/−*^ mice^44^. Furthermore, the results of a recent report indicated that Ppt1 itself undergoes palmitoylation on Cys-6, and this modification regulates its membrane localization and enzymatic activity^45^. Interestingly, it was found that the palmitoylated Ppt1 protein is detected in both cell lysates and in the culture medium, suggesting that a portion of Ppt1 pool is secreted. Since palmitoylation promotes membrane affinity, protein–protein interaction and intracellular trafficking, it is possible that palmitoylated Ppt1 may colocalize with palmitoylated V0a1 on the cytosolic face of the lysosomal membrane and catalyse V0a1 depalmitoylation. In addition, the results of our cell fractionation studies at least in part also support the notion that Ppt1 enzymatic activity is detectable both in the lumen as well as in the membrane fractions (Fig.7a).

### Colocalization of V0a1 with Ppt1 protein

So far, our results indicate that V0a1 palmitoylation is essential for its sorting and trafficking to the lysosomal membrane, and the lack of Ppt1 impairs these processes disrupting lysosomal acidification in Cln1^*−*/*−*^ mice. Since Cln1-encoded Ppt1 is a depalmitoylating enzyme, it is possible that it catalyses depalmitoylation of S-acylated V0a1, which would require colocalization of Ppt1 with V0a1. To delineate whether V0a1 on lysosomal membrane colocalized with Ppt1, we first performed confocal microscopy using V0a1 and Ppt1 antibodies. The results showed that V0a1 immunofluorescence in WT neurons colocalized with that of Ppt1 (Fig. 7b). To confirm these results, we also performed proximity ligation assay^46^ using V0a1 and Ppt1 antibodies, and the results showed strongly positive colocalization signals in WT neurons but not in *Cln1*^*−/−*^ neurons (Fig. 7c). These results suggested that membrane-associated Ppt1 may colocalize with V0a1, and these two proteins may be in close enough proximity to catalyse V0a1 depalmitoylation. Further, the results of the pull-down assay in lysates from HEK-293 cells transfected with Flag-V0a1, Cys25Ser, Cys25Ala or Cys58Ala mutant of V0a1 using Ppt1 antibody showed an interaction of V0a1 with Ppt1, which was lacking in cells transfected with Cys25Ser or Cys25Ala mutant of V0a1, but was unaffected upon mutation of a distant Cysteine residue (Fig. 7d).

An alternative possibility is that there is crosstalk between the cytosolic thioesterase, Apt1 or other recently characterized thioesterases^47^ and lysosomal thioesterase, Ppt1. Such crosstalk, in *Cln1*^*−/−*^ mice lacking Ppt1 in the lysosomal lumen, may downregulate the localization and activity of Apt1 on endosomal/lysosomal membrane. This defect may impair sorting and trafficking of V0a1 to the lysosomal membrane. Another alternative and more general possibility may be that v-ATPase missorting and targeting to the lysosomal membrane is somehow a secondary consequence of lysosomal accumulation of undegraded S-acylated protein cargo due to impaired degradative capability of the lysosomes.

Previously, we reported that Apt1 itself undergoes S-palmitoylation for membrane anchorage, and this enzyme catalyses its own depalmitoylation promoting its cytosol–membrane shuttling required for its function^48^. It is possible that Ppt1 deficiency and consequent accumulation of S-acylated proteins in the lysosomal lumen are monitored via a sensing mechanism similar to the ‘inside-out model' recently proposed for lysosomal amino acids^49^.

### A thioesterase-mimetic restores near-normal lysosomal pH

**Figure 8:**
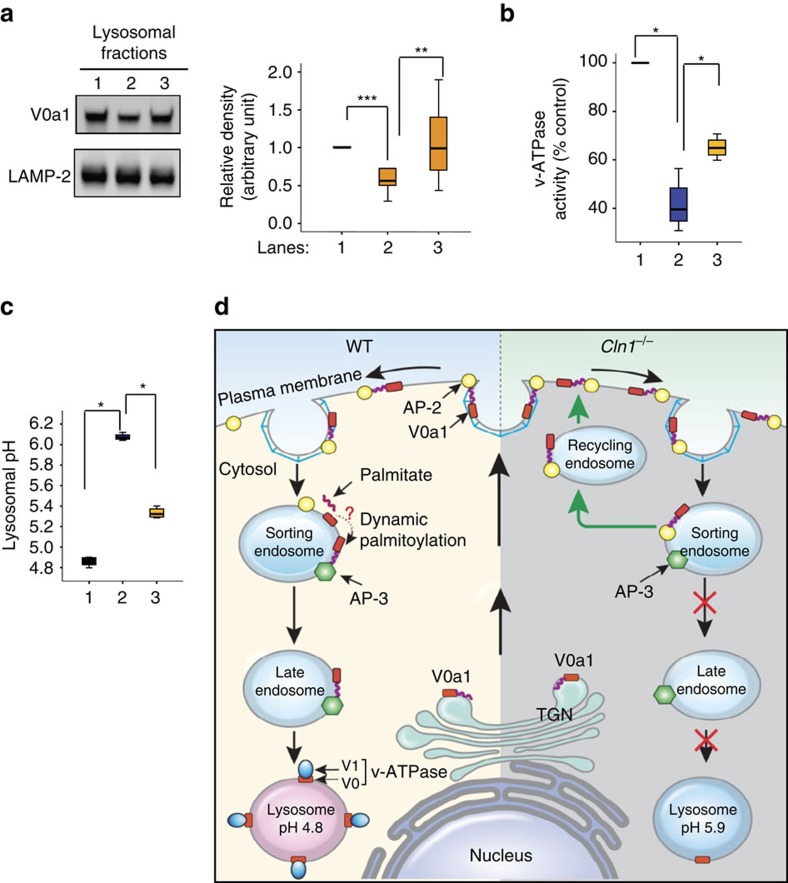
Restoration of near-normal lysosomal pH by NtBuHA in *Cln1*^*−/−*^ mice and schematic explaining how V0a1 may be misrouted to plasma membrane in *Cln1*^*−/−*^ mice. (**a**) Western blot analysis and densitometric quantitation to show that NtBuHA treatment of *Cln1*^*−/−*^mice restores near-normal level of V0a1 in brain, WT (lane 1) versus untreated *Cln1*^*−/−*^ (lane 2), ***P<0.001 and untreated *Cln1*^*−/−*^ (lane 2) versus NtBuHA-treated *Cln1*^*−/−*^ (lane3), **P<0.01, *n*=8. (**b**) v-ATPase activity in isolated lysosomal fractions from WT (lane1), untreated (lane 2) and NtBuHA-treated (lane 3) *Cln1*^*−/−*^mouse brain, WT (lane1) versus untreated *Cln1*^*−/−*^ (lane 2), *P<0.05 and untreated *Cln1*^*−/−*^ (lane 2) versus NtBuHA-treated *Cln1*^*−/−*^ (lane3), *P<0.05, *n*=4. (**c**) Primary neuronal cells isolated from *Cln1*^*−/−*^mouse brain were treated with a thioesterase-mimetic, NtBuHA, for 7 days and lysosomal pH was measured with Oregon green-dextran and TMR dextran, WT (lane1) versus untreated *Cln1*^*−/−*^ (lane 2), *P<0.05 and untreated *Cln1*^*−/−*^ (lane 2) versus NtBuHA-treated *Cln1*^*−/−*^ (lane3), *P<0.05, *n*=4. (**d**) Schematic representation of endosomal sorting and trafficking of V0a1 in WT (left panel) and *Cln1*^*−/−*^ (right panel) mice. Note that in *Cln1*^*−/−*^ cells the V0a1 fails to dissociate from AP-2, preventing it from interacting with AP-3, which is required for its transport from the sorting endosome to the late endosomal/lysosomal membrane; consequently, the V0a1–AP-2 complex is misrouted via recycling endosome to the plasma membrane. This defect impairs lysosomal v-ATPase activity, thereby dysregulating lysosomal acidification in neurons of *Cln1*^*−/−*^ mice.

The results of our study revealed that Ppt1 deficiency in *Cln1*^*−/−*^ mice disrupted endosomal sorting and trafficking of subunit V0a1 of v-ATPase, which was misrouted to the plasma membrane instead of the lysosomal membrane. Since reversible assembly of V1/V0 is necessary for v-ATPase activity, it is possible that S-palmitoylation of V0a1 facilitates the reversible assembly of V1/V0 on lysosomal membrane to regulate lysosomal acidification^9^,^10^,^11^. We found that in *Cln1*^*−/−*^ mice Ppt1 deficiency disrupted endosomal transport of V0a1 to the lysosomal membrane and caused it to be misrouted via recycling endosome to the plasma membrane. This defect caused deficiency of v-ATPase activity on lysosomal membrane, thereby dysregulating lysosomal acidification. To further confirm that thioesterase (that is, Ppt1) deficiency dysregulated lysosomal acidification, we treated *Cln1*^*−/−*^ mice with NtBuHA^50^, a non-toxic derivative of hydroxylamine (HA), which cleaves the thioester linkage in palmitoylated proteins^51^. We found that this treatment substantially increased V0a1-protein levels in lysosomal fractions (Fig. 8a), markedly elevated v-ATPase activity (Fig. 8b) and restored near-normal acidic pH (untreated: 6.07±0.03 versus NtBuHA-treated: 5.33±0.05; *P*<0.05; Fig. 8c). Taken together, these results demonstrated that thioesterase (Ppt1) deficiency in *Cln1*^*−/−*^ mice most likely disrupted V0a1 sorting and trafficking to the lysosomal membrane, thereby impairing v-ATPase activity and dysregulation of lysosomal acidification.

## Discussion

A question, fundamental to understanding the mechanism(s) underlying the LSDs in which neurodegeneration is often the devastating manifestation, is how lysosomal acidification is impaired in these diseases. Dysregulated lysosomal acidification not only occurs in virtually all LSDs^3^,^4^ but also in common neurodegenerative disorders like Alzheimer's^6^,^7^ and Parkinson's^8^. Despite our knowledge that v-ATPase regulates lysosomal acidification^9^,^10^,^11^, how the function of this cellular proton pump is adversely affected by mutations in distinct genes, which lead to various LSDs, remains poorly understood. In this study, using *Cln1*^*−/−*^ mice, a reliable mouse model of INCL, we show that lysosomal acidification is defective in neurons of these mice, which is a likely consequence of misrouted V0a1 subunit of v-ATPase. We also present evidence that membrane-anchored V0a1 undergoes S-palmitoylation on Cys-25 and demonstrate that in Ppt1-deficient *Cln1*^*−/−*^ mice, the levels of V0a1 on lysosomal membrane were significantly lower compared with those in WT counterparts. Notably, we show that the lysosomal acidification defect in *Cln1*^*−/−*^ mice can be chemically rescued by treating these mice with a thioesterase (Ppt1)-mimetic small molecule, NtBuHA, suggesting that the defective acidification phenotype is the result of Ppt1 deficiency. Although Ppt1 is generally considered to be a lysosomal enzyme^38^,^39^, studies demonstrating its presence in extralysosomal locations, especially in neurons, have been reported^40^,^41^,^42^,^43^. On the basis of the results of our experiments, we propose a model that shows that YCCV peptide in the tyrosine motif of V0a1 can be readily docked into the hydrophobic groove in Ppt1 (Supplementary Fig. 6), which harbours the active site of this thioesterase^52^. This may suggest that S-acylated Cys-25 may interact with the Ppt1 catalytic site in the hydrophobic core and facilitate the depalmitoylation of V0a1. Moreover, we show that Ppt1 deficiency in *Cln1*^*−/−*^ mice impairs sorting and trafficking of this critical v-ATPase subunit to the lysosomal membrane, which most likely interferes with V0/V1 assembly dysregulating lysosomal acidification. Taken together, these results support the hypothesis that S-palmitoylation plays critical roles in the endosomal sorting and delivery of V0a1 to the lysosomal membrane and provide insight into a fundamentally important cellular function (that is, the regulation of lysosomal acidification) and an unexpected role of Ppt1 in this process.

Acidification of intracellular organelles plays pivotal roles in regulating vesicular trafficking, endocytosis, autophagy, lysosomal degradation and neurotransmission^2^,^9^,^10^,^11^. It should be noted that variability of pH optima in lysosomal hydrolytic enzymes has been previously reported^53^. More recently, it has been shown that the position of the lysosome within the cell may determine its luminal pH^54^. Lysosomal acidification is dysregulated in virtually all LSDs^2^,^3^,^4^, in the majority of which neurodegeneration is a devastating manifestation^55^. This defect is also reported in common neurodegenerative disorders like Alzheimer's^6^,^7^ and Parkinson's^8^. However, despite intense studies, the mechanism underlying dysregulation of lysosomal acidification in the LSDs and in common neurodegenerative disorders remains largely unclear. In this study, we uncovered a possible mechanism underlying the defective lysosomal acidification in a mouse model of a devastating neurodegenerative LSD, INCL, which is caused by the deficiency of a lysosomal depalmitoylating enzyme, PPT1. Overall, our results demonstrate that S-palmitoylation of V0a1 in WT mice not only facilitates its sorting and steady-state localization on lysosomal membrane but may also allow reversible V1/V0 assembly essential for maintaining v-ATPase activity (Fig. 8d, left panel). However, it appears that the lack of Ppt1 activity in *Cln1*^*−/−*^ mice impaired V0a1 sorting in early endosome, which may have disrupted the dissociation of the V0a1–AP-2 complex preventing V0a1–AP-3 interaction, essential for the transport of V0a1 to the late endosomal/lysosomal membrane. We speculate that in the neurons of *Cln1*^*−/−*^ mice lack of Ppt1 activity causes the V0a1–AP-2 complex to be misrouted via recycling endosome to the plasma membrane (Fig. 8d, right panel) leading to v-ATPase deficiency on lysosomal membrane, thereby dysregulating acidification. Lysosomal acidification is critical for the degradation of endosomal and autophagosomal cargo by lysosomal acid hydrolases^56^ like cathepsin D (CD), which also requires acidic pH for its maturation from pro-CD to enzymatically active CD in this organelle^57^.

Defective acidification of the endosomal/lysosomal system has emerged as one of the major mechanisms underlying pathogenesis of virtually all LSDs and some common neurodegenerative disorders^58^. For example, both *in vitro* and *in vivo* experiments have shown the importance of vesicular acidification in the endocytic transport and degradation or recycling of the endosomal and autophagosomal cargo delivered to the lysosomal lumen. It has been reported that in presenilin-1-deleted blastocysts, defective lysosomal pH caused substantially increased lysosomal pH dysregulating proteolysis^6^. Moreover, dysregulated acidification and increased intracellular pH suppressed the activities of enzymes in endomembrane-bound compartments, which disrupted the clearance of protein aggregates. Furthermore, elevated endosomal and lysosomal pH may have global effects on the proteome. This notion is especially applicable to the membrane proteins that rely on this pathway for their regulation and degradation.

Our results uncovered a mechanism by which lysosomal acidification may be dysregulated in a mouse model of a devastating neurodegenerative LSD, INCL. These results also suggest that varying factors adversely affecting the endosomal transport, lysosomal targeting and assembly of V1/V0 sectors of v-ATPase may dysregulate lysosomal acidification in other LSDs and common neurodegenerative disorders like Alzheimer's and Parkinson's. It has been proposed that therapeutics designed to restore lysosomal function may protect the central nervous system^59^. While the mechanism(s) of altered lysosomal pH in the LSDs until now remained unclear, efforts are already being made to develop strategies to lower the pH in this organelle in order to improve the lysosomal degradative function. Indeed, it has been recently demonstrated that lysosomotrophic acidic nanoparticles restored lysosomal acidic pH, which improved the degradative function of this organelle^60^. Our results have shown that a thioesterase-mimetic small molecule^50^, NtBuHA also improved the endocytic transport of the V0a1 subunit of the v-ATPase, which increased its enzymatic activity and restored near-normal lysosomal acidic pH in *Cln1*^*−/−*^ mice. Clearly, non-toxic, nucleophilic small molecules mimicking thioesterase activity and nanoparticles, which readily cross the blood–brain barrier and ameliorate the defective lysosomal acidification may have therapeutic potential for all LSDs and some common neurodegenerative diseases.

Neurodegeneration is a devastating manifestation in the majority of >50 LSDs^55^. In virtually all LSDs^2^,^3^,^4^ and in some common neurodegenerative diseases like Alzheimer's^6^,^7^ and Parkinson's^8^ dysregulation of lysosomal acidification contributes to pathogenesis. This is because the degradative activity of lysosomal hydrolases requires acidification of the lysosomal lumen for optimal function. While the acidification of intracellular organelles, especially the lysosomes, is of pivotal importance for intracellular digestion, it may also be important in lysosomal nutrient-sensing requiring v-ATPase for cellular homeostasis^49^. We propose that varying factors adversely affecting lysosomal targeting, V1/V0 assembly and/or the function of v-ATPase may also dysregulate lysosomal acidification not only in the neurodegenerative LSD model we used for our study but also in other LSDs and common neurodegenerative disorders.

## Methods

### Chemicals

The following chemicals were purchased from Sigma-Aldrich (St Louis, MO): ammonium chloride (Cat #A9434), phosphoenol-pyruvate (Cat#10108294001, Roche), pyruvate kinase/lactic dehydrogenase (PK/LDH; Cat#P0294), NADPH (Cat#NADPH-RO), ouabain (Cat#O3125) and concanamycin A (Cat#C9705). B27 supplement (Cat#17504-044) and glutaMAX (Cat#35050) were purchased from Invitrogen. *N*-[6-(biotinamido) hexyl]-3′-(2′-pyridyldithio) propionamide-biotin (Cat#21341) was from Thermo Scientific, Rockford, IL.

### Animals and treatments

*Cln1*^*−/−*^ mice^23^ and the Ppt1 antibody were the generous gift from Dr Sandra L. Hofmann, University of Texas South Western Medical Center, Dallas, TX. All animals were housed in a pathogen-free facility with 12 h light/12 h dark cycles with access to water and food *ad libitum*. The treatment of *Cln1*^*−/−*^ mice with NtBuHA has been described previously^51^. Treatment of the mice with NtBuHA (1 mM in drinking water) was initiated at 3 months of age and continued until they were 6 month of age. The animal procedures were carried out according to an animal study protocol (#10-012) approved by the Eunice Kennedy Shriver National Institute of Child Health and Human Development (NICHD), National Institutes of Health (NIH), Animal Care and Use Committee (ACUC). Animals of both sexes were randomized and used for the treatments, and for each experiment the animals were age- and sex-matched.

### Neuron culture and treatments

Cortical neurons were isolated from E15/17 or early postnatal (P0–P2) pups and plated on poly-D. lysine-coated chamber slides or culture dishes with DMEM containing 10% fetal bovine serum. After 24 h the media was replaced and maintained with Neurobasal medium containing B27 and glutamine (Life Technologies) in a humidified incubator with 5% CO_2_ at 37 °C. The neuronal cells were cultured for a week before fixing them for immunohistological analysis or using them for other assays. The cells were immunostained with neuronal marker β-lll-tubulin to confirm that they are neurons. For lysosomal acidification experiments, neurons were pretreated with NH_4_Cl (2 mM) for 1 h or NtBuHA (1 mM) for 7 days. Media were replaced with fresh NtBuHA-containing medium every 12 h.

### Determination of lysosomal pH

For imaging of lysosomal pH-dependent fluorescence the neurons were labelled with 1 μM pH-sensitive lysosensor green DND-189 (L-7535, Life Technologies-Thermo Fisher Scientific) for 10 min, washed with PBS (two to three times) and imaged in the fluorescein isothiocyanate channel. DND-189 fluorescence is inversely proportional to the pH of the lysosome.

To determine and quantitate the lysosomal pH, we used Oregon green dextran (Life Technologies, D-7172) and TMR dextran (D-1819; Life Technologies (Thermo Fisher Scientific), as previously reported^24^ with minor modifications. The ratio of fluorescence from pH-sensitive Oregon green (ex-496, em-524) and pH-insensitive TMR (ex-555, em-580) allowed the measurement of lysosomal pH. For labelling experiments, the primary neuronal cells were grown in neurobasal media for 1 week and then incubated with 1 mg ml^−1^ Oregon green-dextran and 1 mg ml^−1^ TMR dextran in the media for 1 h. The dextran-containing media were then replaced with media without dextran and chased for 2.5–3 h to allow the dextran to accumulate in late endosomal/lysosomal compartment. The labelled cells were then harvested, washed three to four times in PBS and then resuspended in MES calibration buffer solution of varying pH (3.5–7.5) containing 5 mM NaCl, 115 mM KCl, 1.2 mM MgSO_4_ and 25 mM MES in the presence of 10 μM nigericin and 10 μM monencin. The ratio of the intensities of green fluorescence versus red fluorescence emission was plotted against pH to generate a standard curve against which the pH in WT and *Cln1*^*−/−*^ neurons was determined. The WT and *Cln1*^*−/−*^ neuronal cells were loaded with the pH sensors and resuspended in the same calibration buffer (pH 7.5) without nigericin and monencin for measuring the pH.

### Lysosome purification

Lysosomes were purified from mouse brain cortex with Optiprep density gradient media using the lysosome isolation kit (LYSISO1, Sigma-Aldrich) as described in the supplier's protocol with minor modifications as follows: freshly isolated cortical tissues were homogenized in 4 volumes of 1 × extraction buffer containing Halt Protease Inhibitor (PI) Cockail (Thermo Fisher; Cat#78430) and centrifuged for 1,000*g* for 10 min. The supernatant was then centrifuged at 20,000*g* for 20 min at 4 °C. The resultant pellet containing the crude lysosomal fraction was resuspended in 1 × extraction buffer and adjusted to 19% Optiprep and layered over 22.5% Optiprep in a multistep Optiprep gradient consisting of 27, 22.5, 19, 16, 12 and 8% Optiprep according to the manufacturer's protocol and centrifuged for 4 h at 150,000*g* in a swing out rotor. The various layers, F1 (top layer) to F5 (bottom layer), formed at the junction of each gradient were collected after centrifugation, and the enrichment of lysosomes in a fraction was confirmed by measuring β-*N*-acetylglucosaminidase activity and detection of lysosomal membrane marker (LAMP 1) through immunoblotting. Fraction 2 and fraction 3 were found to be lysosome-enriched and were combined for all assays and immunoblotting experiments.

### v-ATPase enzyme activity assay

Freshly isolated lysosomal fractions were used to measure the v-ATPase activity by an ATP/NADPH-coupled assay^61^. Briefly 20–50 μg of lysosomal protein was thoroughly mixed and incubated in 1 ml of reaction buffer containing 25 mM TEA (triethanolamine) pH 7.4, 2 mM ATP, 100 μg ml^−1^ BSA, 3 mM phosphoenol-pyruvate, 20 U pyruvate kinase/lactic dehydrogenas, 0.2–0.4 mg ml^−1^ NADPH (from freshly prepared stock of 20 mg ml^−1^), 2 mM dithiothreitol, 2 mM Ouabain (inhibits Na^+^, K^+^-ATPase), 5 mM Na Azide (inhibits the mitochondrial ATPase) in presence or absence of 1 μM Concanamycin A (specific inhibitor of v-ATPase) for 30 min at 37 °C. A volume of 285 μl of the sample was added to 96-well plates. The baseline absorbance of NADPH was recorded for 3–5 min at 340 nm in the Synergy HTX. The reaction was started by addition of 15 μl of 260 mM Mg Acetate, and the decrease in NADPH absorbance was recorded for 15 min. The concanamycin A-sensitive ATPase activity normalized with lysosomal protein was taken as a measure of v-ATPase activity.

### Mutagenesis of V0a1

FLAG-V0a1 and GFP-V0a1 constructs were purchased from Genecopoeia. To generate palmitoylation site mutants, the cysteine residues in positions 24 and 25 were mutated to serine. For mutating the tyrosine and dileucine motifs the tyrosine residue 23 was mutated to alanine or valine; leucine residue 147 to alanine and leucine residue 148 to alanine. These mutants were generated by PCR using the Quik Change site-directed Mutagenesis Kit from Agilent Technologies by the supplier (Bioinnovatise, Rockville, MD). The mutations were confirmed by direct DNA sequencing. The primers used for mutagenesis are: V0a1-Cys24Ser: Forward-5′-ctttttctacagtcagaggctgcttatTCTtgtgtcagtgaattaggagaacttgg; Reverse-3′-CCAAGTTCTCCTAATTCACTGACACAAGAATAAGCAGCCTCTGACTGTAGAAAAAG; V0a1-Cys25Ser:Forward-5′-ctacagtcagaggctgcttattgtTCTgtcagtgaattaggagaacttggaaagg; Reverse-3′-CCTTTCCAAGTTCTCCTAATTCACTGACAGAACAATAAGCAGCCTCTGACTGTA G.V0a1-Tyr23Ala:Forward-5′-gcccagctatttctacagtctgaagctgctGCTtgctgtgtcagtgaattaggagagc; Reverse-3′-GCTCTCCTAATTCACTGACACAGCAAGCAGCAGCTTCAGACTGTAGAAATAGCTGGGC; V0a1-Tyr23Val: Forward-5′-gcccagctatttctacagtctgaagctgctGTTtgctgtgtcagtgaattaggagagc; Reverse-3′- GCTCTCCTAATTCACTGACACAGCAAACAGCAGCTTCAGACTGTAGAAATAGCTGGGC; V0a1-Cys24Ala: Forward-5′-cagctatttctacagtctgaagctgcttatGCCtgtgtcagtgaattaggagagcttggg; Reverse-3′-CCCAAGCTCTCCTAATTCACTGACACAGGCATAAGCAGCTTCAGACTGTAGAAATAGCTG; V0a1-Cys58Ala:Forward-5′-gaggaaatttgtgaatgaagttagaagaGCTgaagaaatggatcgaaaactccgatttgt; Reverse-3′-ACAAATCGGAGTTTTCGATCCATTTCTTCAGCTCTTCTAACTTCATTCACAAATTTCCTC; V0a1-leu147Ala: Forward-5′-gtttttcgatgagatggcggatccagacGCGttggaagagtcctcatcactcttgg; Reverse-3′-CCAAGAGTGATGAGGACTCTTCCAACGCGTCTGGATCCGCCATCTCATCGAAAAAC;V0a1-leu148Ala: Forward-5′-cgatgagatggcggatccagacctgGCGgaagagtcctcatcactcttggagc; Reverse-3′-GCTCCAAGAGTGATGAGGACTCTTCCGCCAGGTCTGGATCCGCCATCTCATCG.

### Transfection of HEK-293 cells with cDNA constructs

HEK-293 cells were transfected with cDNA constructs or shRNA constructs using Lipofectamine LTX reagent (Invitrogen) according to the manufacturer's protocol. The plasmids used for transfection were prepared using a Plasmid Midi Kit (Qiagen).

### ABE assay

The ABE method^29^ was used to determine the palmitoylation status of V0a1 and V0a2 with minor modifications. Briefly, HEK-293 cells transfected with V0a1–cDNA or V0a2-cDNA or V0a1-M-1 or V0a1-M-2 cDNA constructs were lysed with RIPA buffer (Pierce), and the lysates were incubated with 10 mm *N*-ethylmaleimide (NEM; Thermo Scientific, 23030) for 1 h at 4 °C. The samples were subjected to one chloroform/methanol (CM) precipitation and resuspended in solubilizing buffer containing 10 mM NEM and incubated overnight in presence of PI at 4 °C with gentle mixing. NEM was then removed by three sequential CM precipitations. Following the third CM precipitation, the protein was solubilized in solubilizing buffer and was divided into two equal aliquots. One aliquot was treated with 1 M HA (Sigma-Aldrich), pH 7.4 in presence of 1 mm *N*-[6-(biotinamido) hexyl]-3′-(2′-pyridyldithio) propionamide-biotin (HPDP-Biotin), 0.2% Triton X-100 (Sigma-Aldrich) and PI. The second aliquot was treated with identical mixture but without HA. After 1 h of incubation at room temperature the proteins were precipitated by CM method and treated with 200 μM HPDP-biotin, 0.2% Triton X-100 and PI at room temperature for 1 h. HPDP*-*biotin was then removed by three sequential CM precipitations. Following the third precipitation, proteins were solubilized and an aliquot from each sample was kept as loading control before immunoprecipitation with streptavidin–agarose (Cat #20353, Thermo Scientific). The immunoprecipitates were resolved by SDS–PAGE under denaturing and reducing conditions. Samples were then subjected to immunoblotting using FLAG-antibody (F-3165, Sigma-Aldrich, Dilution 1:1,000).

### Plasma membrane isolation

For isolation of pure plasma membrane free of lysosomal contamination, a combination of differential centrifugation and gradient centrifugation was used as previously reported^62^. Briefly, the cortical tissues were homogenized in 4 volumes of homogenizing media (0.32 M sucrose, 1 mM EDTA, 10 mM HEPES) with a Dounce homogenizer. The homogenates were centrifuged at 1,000*g* for 10 min to pellet the nuclear fraction followed by centrifugation of the resultant supernatant at 3,000*g* for 10 min to pellet the heavy mitochondrial fraction. The resultant supernatant was then centrifuged at 15,000*g* for 20 min to pellet down majority of the lysosomal fraction. The supernatant was then centrifuged at 100,000*g* for 1 h. The 100,000*g* pellet was resuspended in homogenizing media and layered over a discontinuous (20, 15 and 10%) Optiprep gradient. After 1 h of centrifugation at 100,000*g*, the plasma membranes were collected from the 10% Optiprep layer.

### Bromopalmitate treatment of cultured brain slices

Brain tissues isolated from P2–P6 pups were sliced (thickness: 0.5 mm) with brain slicer (ZIVIC Instruments) under sterile conditions, and the slices were placed on cell strainers in six-well plates containing DMEM (supplemented with 10% FBS) in a way that the brain slices were in contact with the media but not submerged in it. For bromopalmitate treatment, the slices were incubated in media containing dimethylsulphoxide (for control) or 50 μM bromopalmitate for 12 h. Following incubation the slices were gently rinsed with PBS and processed for the assays.

### Immunoprecipitation

Mouse brain cortical tissues were homogenized in 4 volumes of homogenizing media (0.32 M sucrose, 1 mM EDTA, 10 mM HEPES with PI cocktail) with a Dounce homogenizer. The homogenates were centrifuged at 1,000*g* for 10 min to pellet the nuclear fraction. The supernatant (post-nuclear fraction) was solubilized in 1 volume of RIPA buffer followed by centrifugation at 10,000*g*. The resultant supernatant was diluted to 0.5–1 mg ml^−1^ protein with PBS containing 0.5% NP-40 for immunoprecipitation reaction. The HEK-293 cells were lysed in RIPA buffer by passing through the 27-gauge insulin syringe for 10–12 times and centrifuged at 10,000*g* for 10 min. The soluble supernatant was diluted in PBS containing 0.5% NP-40 for immunoprecipitation reaction. The samples were pre-cleared and incubated with V0a1 antibody (Abnova; H00000535-A01 Lot#11257; dilution 1:500 and Novus; NBP1-89342; Lot#B96917; dilution 1:500), AP-2 antibody (BD Biosciences; 610502; dilution 1:1,000), AP-2 antibody (Abcam; ab189995; dilution 1: 500), AP-1 antibody (BD Biosciences; 610386; dilution 1:1,000), AP-3 antibody (Proteintech, 13384-1-AP, dilution 1:200), PPT1 antibody^23^ (dilution 1:1,000), overnight at 4 °C. Rockland true blot ip-specific beads (50 μl) were then added and the mixtures were incubated for 1 h on a rocker platform at 4 °C. The beads were then washed extensively with PBS and resuspended in 40 μl of 2 × electrophoresis sample buffer (Santa Cruz Biotechnology, sc-24945). Immunoprecipitates were resolved by SDS–PAGE and analysed by western blotting. For detection of the protein bands horseradish peroxidase (HRP)-conjugated secondary antibodies (Santa Cruz Biotechnologies) or true blot secondary antibodies (Rockland Immunochemicals) were used to minimize the detection of heavy and light chains of immunoglobulin.

### Immunocytochemistry and confocal imaging

Primary neuronal cells isolated from mouse brain were cultured on poly-D-lysine-coated chamber slides. The cells were washed two to three times with PBS and fixed using 4% paraformaldehyde or methanol. Paraformaldehyde-fixed cells were permeabilized with 0.1% Triton X-100 in PBS, but the methanol-fixed cells were not permeabilized. After blocking with 10% normal goat serum, the cells were incubated with primary antibodies overnight at 4 °C followed by Alexa Fluor-conjugated secondary antibodies (Life Technologies (Thermo Fisher Scientific) for 1 h at room temperature. When using anti-goat primary antibody the cells were blocked with Image-iT FX signal enhancer (Thermo Fisher Scientific; 136933). The primary antibodies used were V0a1 (Abnova; H00000535-A01 Lot#11257; dilution 1:100), V0a1 (Novus Biologicals, NBP1-89342; Lot#B96917; dilution 1:100), AP-2 (BD Biosciences, 610502; dilution 1:500), AP-2 (Abcam, ab189995; dilution 1:500), AP-3 (DHSB, University of Iowa, SA4, dilution 1:200), LAMP1(Santa Cruz Biotechnology, sc19992, dilution 1:500), LAMP2 (EMD Millipore, MAB40; dilution 1:500; GM-130 (BD Biosciences, 610823; dilution 1:1,000), Calnexin (Cell Signalling, C5C9; dilution 1:100), EEA1 (BD Biosciences, 610457; dilution 1:200), Rab 11(Cell Signalling, D4F5; dilution 1:100), Rab 9 (Cell Signalling, D52G8; dilution 1:100), Na^+^/K^+^-ATPase (Cell Signalling, 3010S; dilution 1:200) and PPT1 antibody^23^ (dilution 1:1,000). For colocalization of AP-2 with Rab11 the cells were first fixed with paraformaldehyde (PFA) and probed with Rab11 antibody and then fixed with methanol again before probing with AP-2 antibody as the two antibodies worked with different fixatives. Cells were mounted using DAPI-Fluoromount G (Thermo Fisher, 010020) and fluorescence was visualized with the Zeiss LSM 710 Inverted Meta confocal microscope (Carl Zeiss). The image was processed with the LSM Image Software (Carl Zeiss). Manders colocalization coefficient was calculated using the Zen Desk Software from Zeiss. Manders' M1 and M2 coefficients were calculated to determine the extent of colocalization between a pair of proteins stained and imaged in two channels (that is, red and green)^63^. Manders M1 determines the degree of green channel colocalizing with red and M2 determines the reverse. We have used the weighted colocalization coefficient for analysis.

### Western blot analysis

For western blot, protein samples (5–20 μg) were resolved by electrophoresis using 4–12% SDS–polyacrylamide gels (Invitrogen) under denaturing and reducing conditions and electrotransferred to nitrocellulose or polyvinylidene difluoride membranes (Invitrogen). The membranes were blocked with 5% nonfat dry milk (Bio-Rad) and then subjected to immunoblot analysis using standard methods. The primary antibodies used for the immunoblots were as follows: V0a1 (Abnova; H00000535-A01 Lot#11257; dilution 1:500), V0a1 (Novus Biologicals, NBP1-89342; Lot#B96917; dilution 1:500), AP-1 (BD Biosciences, 610386; dilution1:1,000), AP-2 (BD Biosciences, 610502; dilution 1:500), AP-3 (DHSB, University of Iowa, SA4, dilution 1:500), LAMP1(Santa Cruz Biotechnology, sc19992, dilution 1:500), FLAG (Sigma-Aldrich, F-3165; Dilution 1:1,000), pan-cadherin (Abcam, ab6528; dilution 1:1,000), β-actin (US Biological), GFP (Abcam, ab290; dilution 1:1,000) and ATP6V1A (Abcam, ab137574; dilution 1:1,000). The blots were then probed with HRP-conjugated secondary antibodies (Santa Cruz Biotechnology) followed by detection using SuperSignal west femto or pico solution (Pierce, Thermo Scientific) according to the manufacturer's instructions. Image quant 4000 mini (GE Healthcare Lifesciences) was used to capture the chemiluminecent signals, and the immunoblots were quantitated using the image quant TL software (GE Healthcare Lifesciences). Experiments were repeated at least three to five times to confirm the reproducibility. Images for all the western blots have been cropped for presentation. Images of the full size blots are presented in Supplementary Fig. 7.

### Cell fractionation and Ppt1 activity assay

Mouse brain cortex was homogenized in 4 volumes of homogenizing buffer with PI. The homogenate was centrifuged at 1,000*g* to pellet the nuclear fraction. The Post-nuclear fraction was centrifuged at 20,000*g* to get the total membrane (P1) and cytosolic fractions (S1). The total membrane fraction was subjected to hypotonic shock by resuspending in 0.03 M sucrose and keeping it on ice for 30 min. The P1 fraction was then centrifuged at 100,000*g* for 1 h to pellet the matrix (P2) and isolate the soluble luminal proteins (S2). The P2 was washed three times in 0.03 M sucrose. At each step the pellets were resuspended in a known volume of buffer to calculate the total amount of protein in each fraction.

PPT1 activity was measured as previously reported^64^. Briefly, all the fractions were further diluted in water and sonicated. The samples (10 μg protein) were incubated with the substrate 4-Methylumbelliferyl-6-thiopalmitoyl-β-D-glucoside (Moscerdam Substrates, the Netherlands) for 1 h at 37 °C as described in the manufacturer's protocol. The reaction was terminated by the addition of Stop Buffer (0.5 M Na_2_CO_3_/NaHCO_3_, pH 10.7, 0.025% Triton X-100). The fluorescence of the product, 4-Methylumbelliferone, was measured with Flexstation2 (Molecular Device).

### Proximity ligation assay

Duolink proximity ligation assay^46^ was used to study interaction between AP-2/V0a1, AP-3/V0a1 and Ppt1/V0a1. Briefly, the primary neurons from WT and *Cln1*^*−/−*^ mice were grown in chamber slides, fixed with paraformaldehyde and permeabilized with 0.1% Triton X-100 in PBS. The cells were blocked with PLA blocking buffer and incubated with V0a1 antibody (Abnova; H00000535-A01 Lot#11257; dilution 1:100), AP-2 antibody (Abcam; ab189995; dilution 1:800), AP-3 antibody (DHSB, University of Iowa, SA4, dilution 1:100) and Ppt1 antibody (Dr Hofmann Laboratory; dilution 1:1,000) overnight. Following three washes with PBS the cells were incubated with anti-rabbit-MINUS (Sigma-Aldrich; DUO92005 for voa1 antibody) and anti-mouse-PLUS (Sigma-Aldrich; DUO92001-for PPT1 antibody) PLA probes and subjected to ligation and amplification reaction using Duolink *In Situ* Detection Reagents Orange (Sigma-Aldrich; DUO80102) or Duolink *In Situ* Detection Reagents Green (Sigma-Aldrich; DUO9201) according to the manufacturer's protocol. For controls, cells were similarly processed but without primary antibody or with one primary antibody only. Absence of PPT1 in *Cln1*^*−/−*^ cells served as additional controls. Fluorescent signals were detected when the primary antibodies were in close proximity (<40 nm distance). The cells were mounted with DAPI-Fluoromount G (Thermo Fisher, 010020) and visualized with Zeiss 710 inverted confocal microscope. Z stack images were taken to include all the signals at different focal planes, and the images were merged using maximum intensity projection. The Duolink image tool was used to count the PLA signals.

### RNA isolation and real-time RT–PCR

Total RNA was isolated from brain cortical tissues of WT and *Cln1*^*−/−*^mice using the RNA easy Mini Kit (QIAGEN) followed by cDNA synthesis using the Superscript III First-Strand Synthesis kit (Invitrogen) according to the manufacturer's instructions. The levels of mRNA expression was quantified and compared by real-time RT–PCR with SYBR Green PCR mix using ABI Prism 7000 Sequence detection system and analysed by using the ABI Prism Software Version 1.01 (Applied Biosystems). The Ct values were calculated using GAPDH as control. The primers used for V0a1 mRNA are: Forward-5′-CTGTTATCCTCGGCATCATCCAC; Reverse-3′-CAGGTAGCCAAACAACGAGGAC.

### shRNA experiments

HEK-293 cells were transfected with scrambled shRNA (GE, Dharmacon, RHS4346) and AP-2 (GE, Dharmacon, V3LMM_503345) using lipofectamine LTX. Cells were harvested after 48 h, and GFP-positive cells were sorted using a FACS Aria Flow cytometer (BD Biosciences). By post-sort analysis, the purity of the population was 98–99% transfected cells. The sorted cells were further cultured in media containing 2 μg ml^−1^ puromycin for 6–7 days to ensure a pure population of stably transfected cells. These stably transfected cells were then grown in antibiotic-free media and collected for the isolation of plasma membrane.

### Statistical analysis

The data are represented as the mean±s.d. and the ‘*n*' numbers represent the number of biological replicates for each experiment. For imaging studies ‘*n*' is the total number of cells analysed, and the data are represented as the mean±s.e.m. We used permutation test based on the mean differences to calculate the *P* values to examine the differences between two independent groups. For small *n*, *P* values from complete enumeration were calculated. A *P* value of less than 0.05 is considered statistically significant. Some of the data has been represented as box plots. The centre line in the box plots is the median, box limits are first and third quartiles and whiskers represent minimum and maximum values.

### Data availability

All data are available from the authors.

## Additional information

**How to cite this article:** Bagh, M.B. *et al*. Misrouting of v-ATPase subunit V0a1 dysregulates lysosomal acidification in a neurodegenerative lysosomal storage disease model. *Nat. Commun.* **8**, 14612 doi: 10.1038/ncomms14612 (2017).

**Publisher's note:** Springer Nature remains neutral with regard to jurisdictional claims in published maps and institutional affiliations.

## Supplementary Material

Supplementary Information Supplementary Figures.
