# Supplementary material for: Misrouting of v-ATPase subunit V0a1 dysregulates lysosomal acidification in a neurodegenerative lysosomal storage disease model
Source: Nat Commun. 2017 Mar 7;8:14612. doi: 10.1038/ncomms14612 (PMC5344305; doi:10.1038/ncomms14612)

Supplementary Figure 1

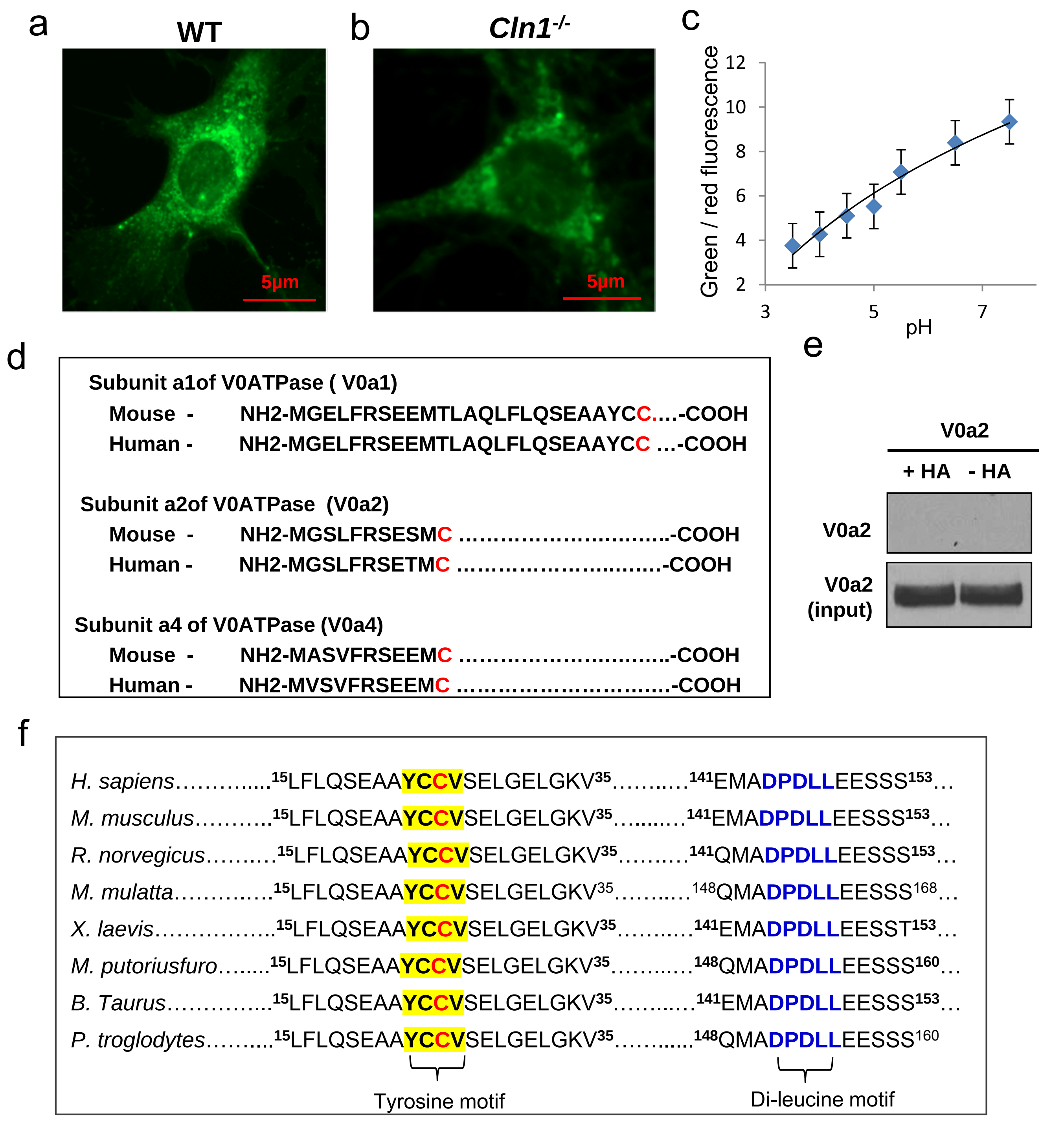

Supplementary Figure 1. Lysosomal pH, predicted palmitoylation sies and tyrosine and di-leucine motifs in V0 subunits of v-ATPase

(**a** & **b**) Lysosomal pH in primary cultures of neurons from of WT and *Cln1*$^{-/-}$ mice, respectively, were visualized by loading the cells with DND-189. (**c**) Calibration curve for the measurement of lysosomal pH was generated by plotting the ratio of fluorescence intensities from cells loaded with pH-sensitive Oregon green-dextran and pH-insensitive TMR-dextran at varying pH as indicated. The data are representative of at least 4 independent experiments. (**d**) Potential palmitoylation site(s) in V0a1, V0a2 and V0a4 predicted by CSS-PALM-4. (**e**) HEK-293 cells were transfected with Flag-V0a2 and cell lysates subjected to ABE assay were analyzed by Western blotting. (**f**) Sequence alignment of V0a1 of eight different species to show that the palmitoylated Cys-25 (red), tyrosine motif (yellow highlight) and the di-leucine motif (blue) are conserved.

Supplementary Figure 2

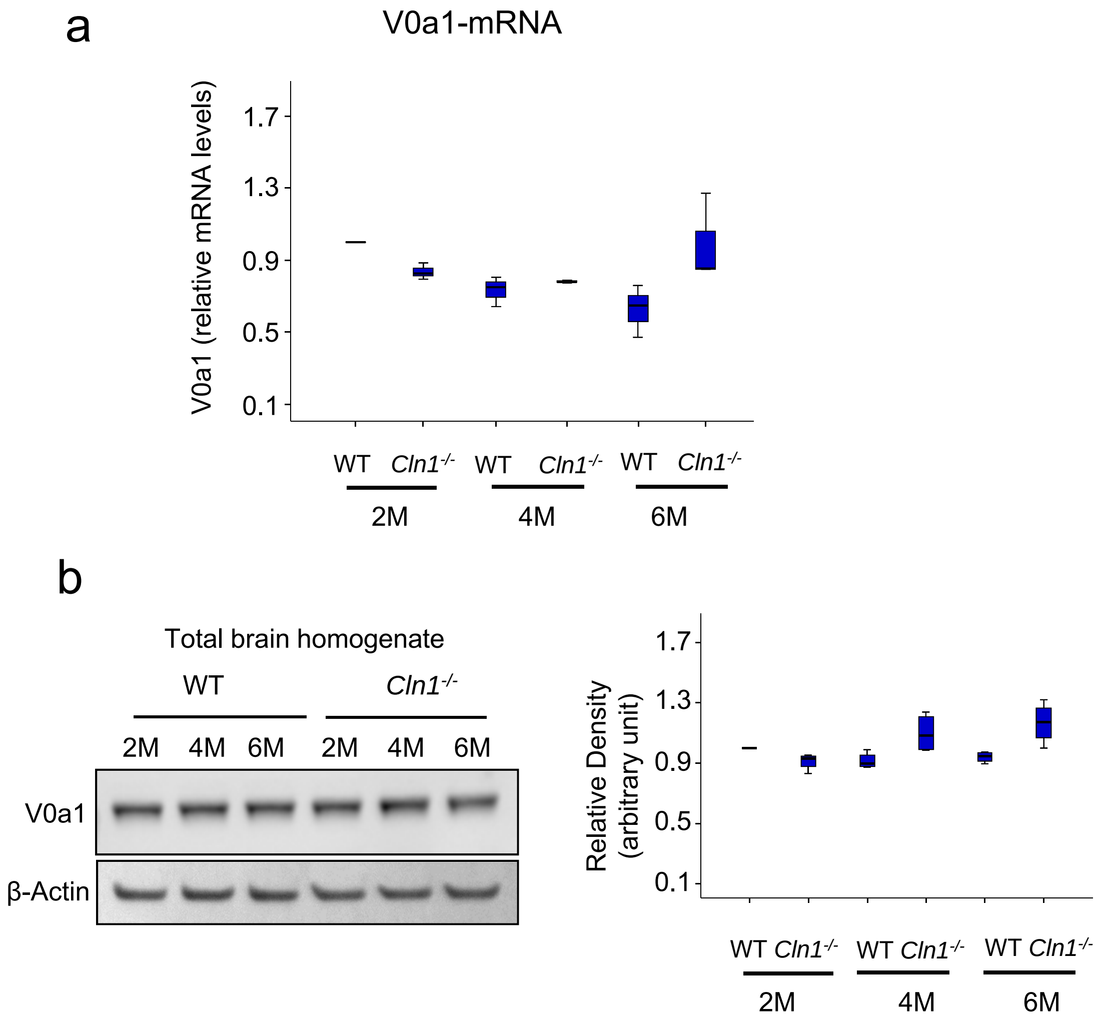

Supplementary Figure 2: Levels of V0a1-mRNA and V0a1-protein levels in WT and *Cln1*$^{-/-}$ mice.

(**a**) V0a1-mRNA levels as quantified by qRT-PCR using total RNA from the cerebral cortices of 2-, 4- and 6-month old WT *and Cln1*$^{-/-}$ mice, n=3 animals per group. (**b**) Western blot analysis and quantitation of total V0a1 in cortical lysates from 2-, 4- and 6-month old WT and *Cln1*$^{-/-}$ mice, (n=4).

Supplementary Figure 3

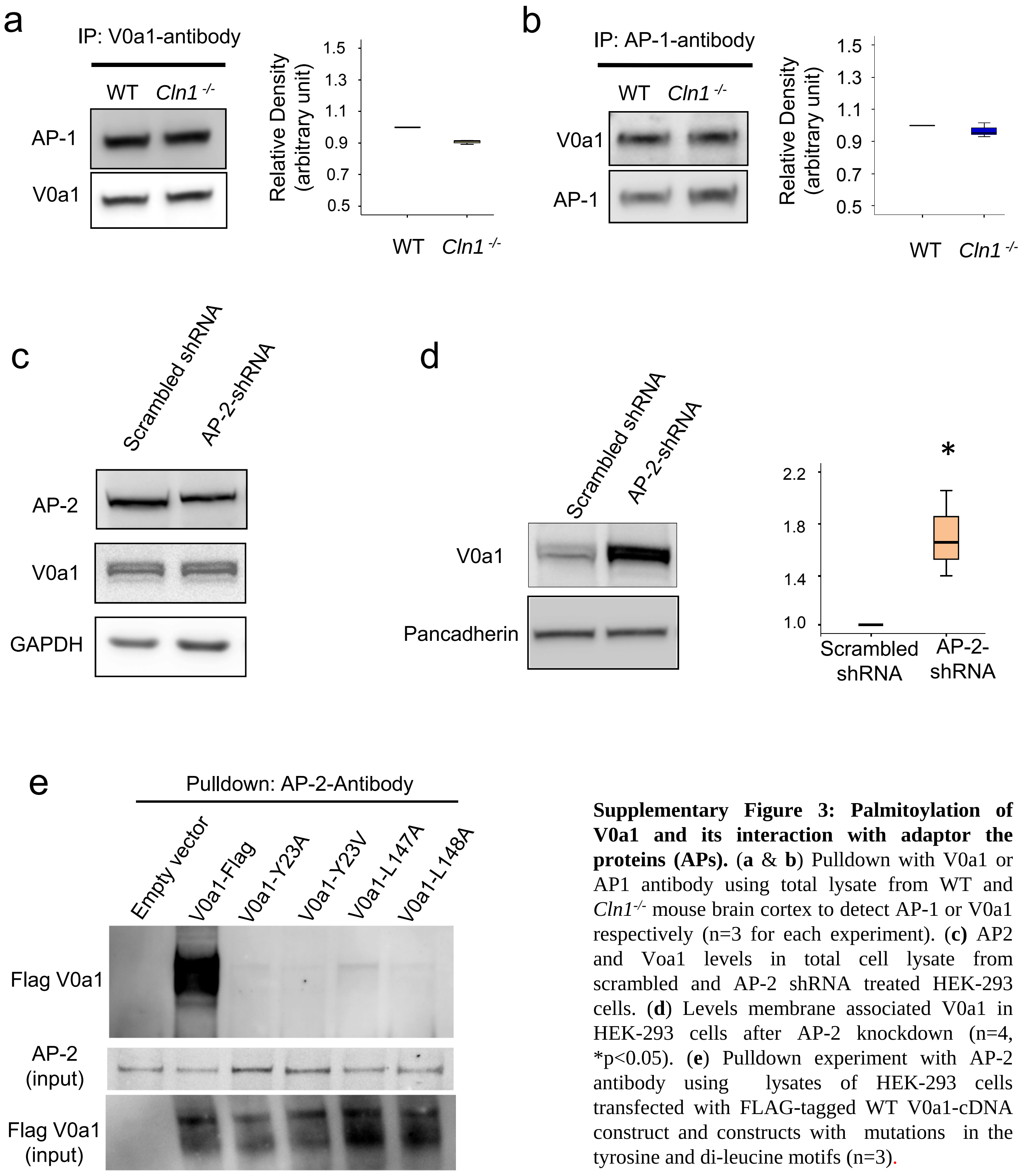

Supplementary Figure 3: Palmitoylation of V0a1 and its interaction with adaptor the proteins (APs). (**a** & **b**) Pulldown with V0a1 or AP1 antibody using total lysate from WT and *Cln1*$^{-/-}$ mouse brain cortex to detect AP-1 or V0a1 respectively (n=3 for each experiment). (**c**) AP2 and Voa1 levels in total cell lysate from scrambled and AP-2 shRNA treated HEK-293 cells. (**d**) Levels membrane associated V0a1 in HEK-293 cells after AP-2 knockdown (n=4, *p<0.05). (**e**) Pulldown experiment with AP-2 antibody using lysates of HEK-293 cells transfected with FLAG-tagged WT V0a1-cDNA construct and constructs with mutations in the tyrosine and di-leucine motifs (n=3).

Supplementary Figure 4

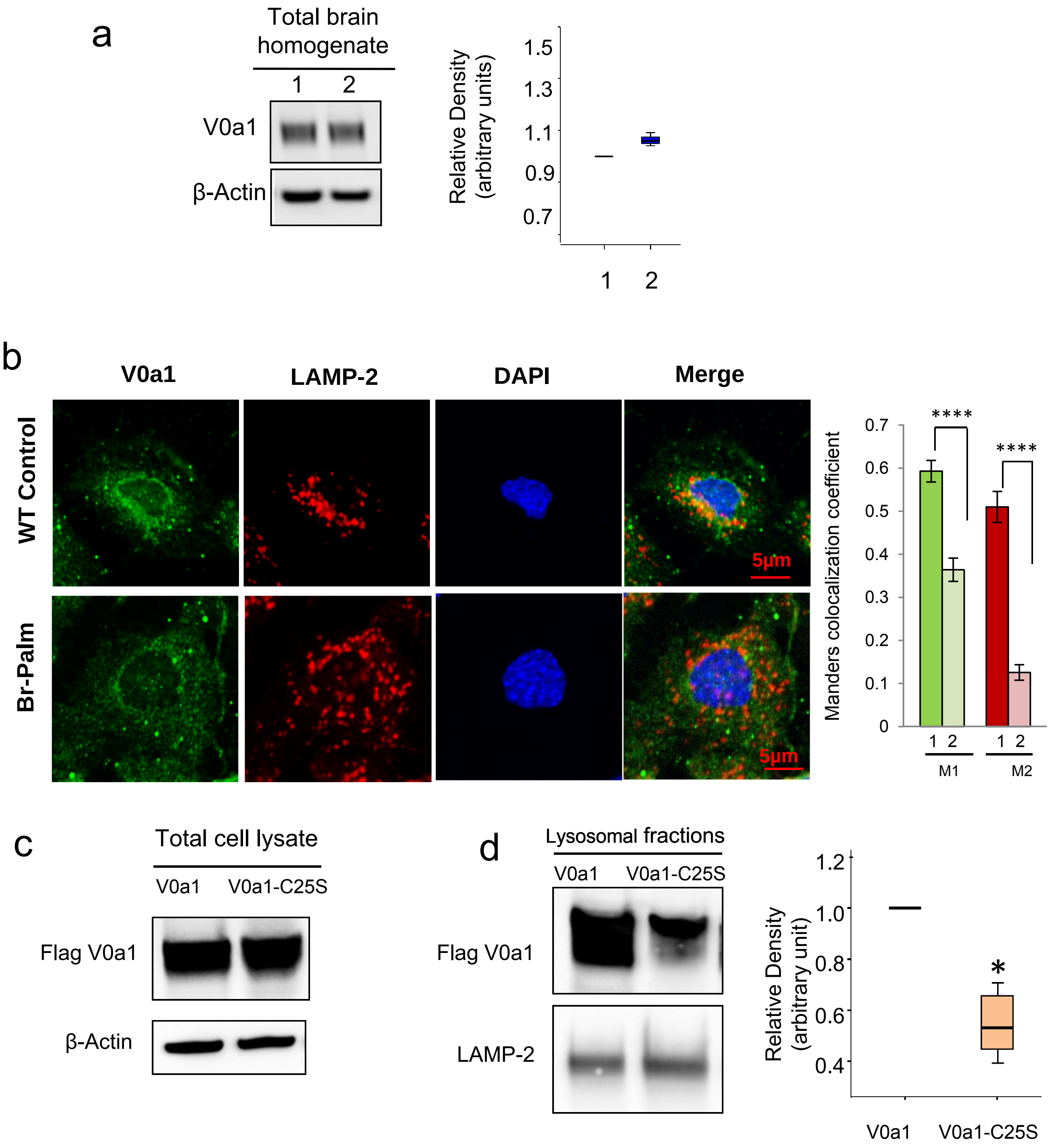

Supplementary Figure 4: Palmitoylation of V0a1 is required for trafficking to lysosomal membrane. (**a**) Western blot analysis to detect V0a1 in total brain lysate from WT brain slices treated with bromopalmitate (lane 2) and untreated control (lane 1)(n=4). (**b**) Lysosomal localization of V0a1 in WT neuronal cells; (1), untreated and (2), treated with bromopalmitate (n=26 for WT and n=21 for *Cln1*$^{-/-}$, ****p<0.0001).(**c**) Total levels of FLAG-tagged WT-V0a1 and C25S--mutant in HEK-293 cells. (d) Lysosomal levels of FLAG-tagged WT-V0a1 and C25S--mutant in HEK-293 cells (n=3, *p<0.05).

Supplementary Figure 5

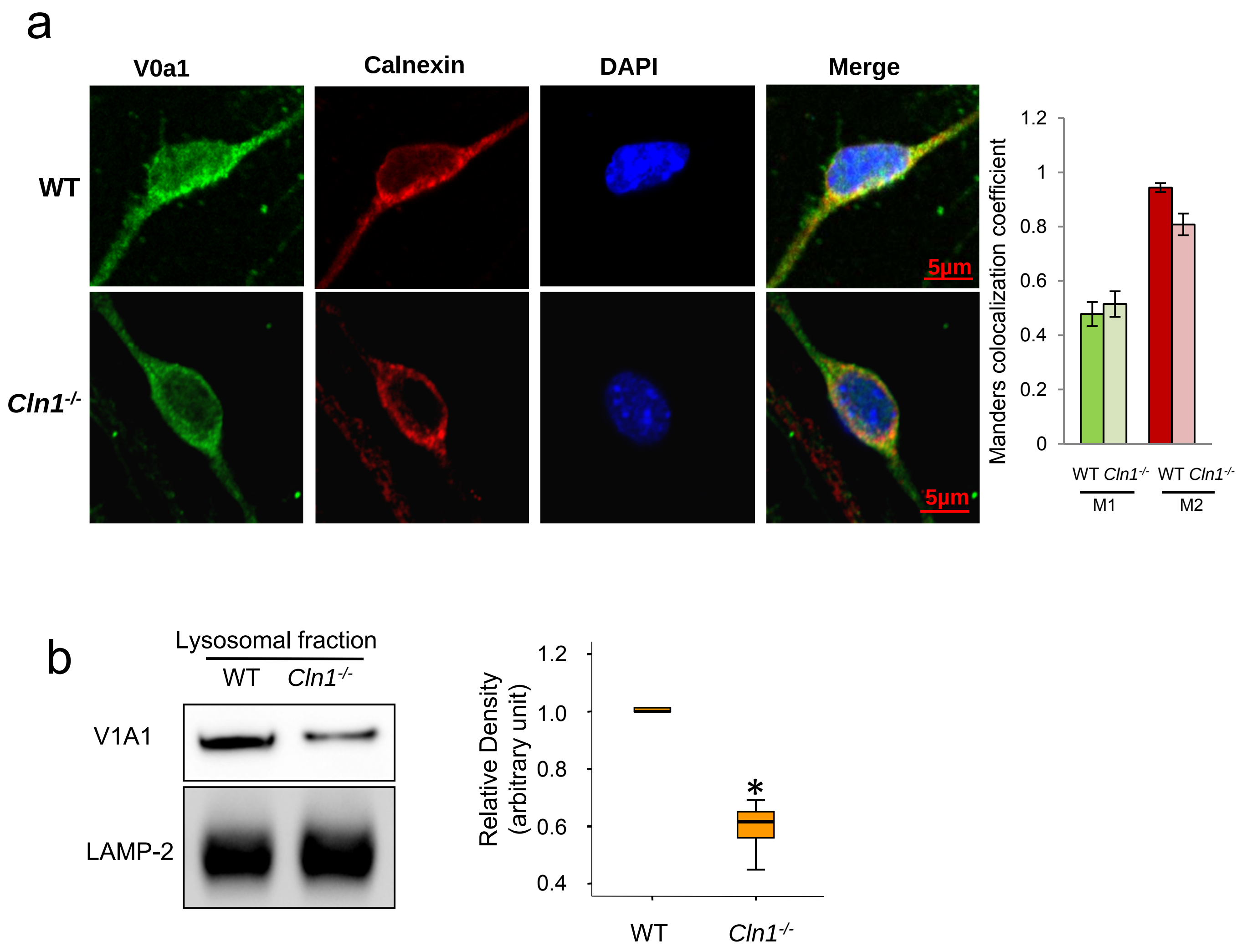

Supplementary Figure 5. **ER-localization of V0a1 and reduced lysosomal V1A1 levels.** (**a**) Confocal imaging colocalizing the ER marker (Calnexin) with V0a1 in cortical neurons from WT and *Cln1*$^{-/-}$ mouse brain(n=7 for WT and 11for *Cln1*$^{-/-}$).(**b**) Lysosomal levels of V1A1 in isolated brain tissues from WT and *Cln1*$^{-/-}$mice. Densitometric quantitation (right panel) is from 4 independent experiments, *p<0.01.

Supplementary Figure 6

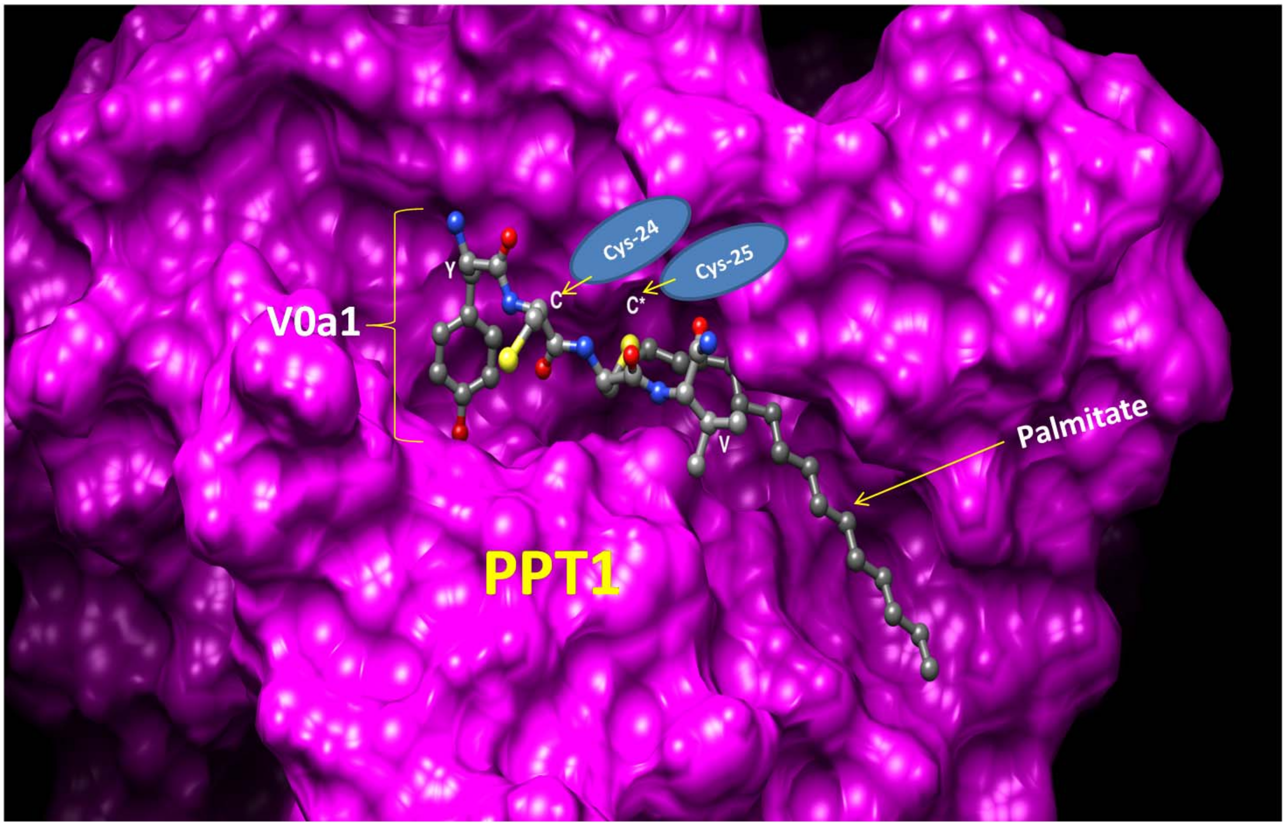

Supplementary Figure 6. **Proposed molecular model to explain how PPT1-mediated catalysis of V0a1-depalmitoylation may occur on lysosomal membrane.** This model is generated by using YCCV peptide in palmitoylated-V0a1 docked into the Ppt1 active site localized in the hydrophobic groove to suggest that S-palmitoylated Cys-25, but not Cys-24, may interact with the catalytic site of Ppt1 to facilitate its depalmitoylation. Crystal structure of Ppt1 was resolved, which demonstrated the presence of the catalytic site in the hydrophobic core through which the substrate are depalmitoylated (see Fig.1 of Ref. 50). Y, Tyrosine; V, Valine; C, Cys-24; C*, Cys-25, which undergoes S-palmitoylation.

Supplementary Figure 7. Original images of immunoblot

Figure 1d

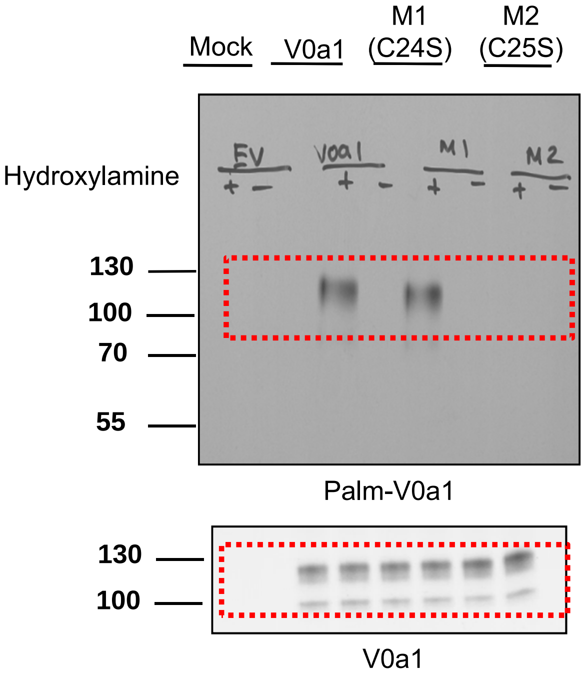

Figure 1e

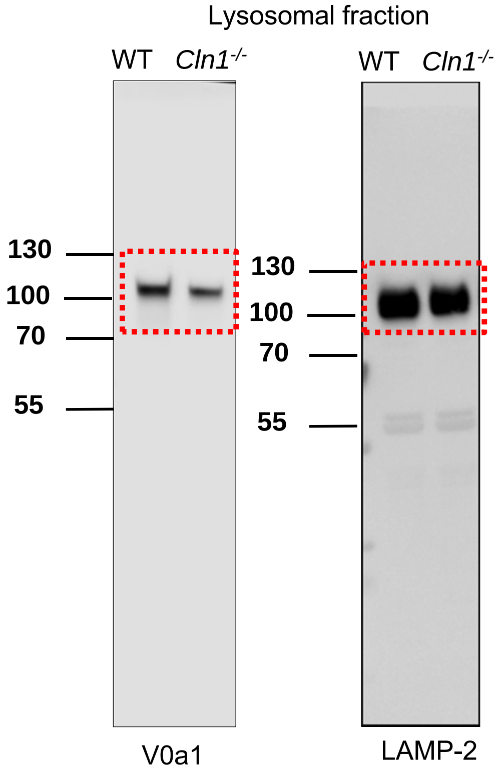

Figure 2a

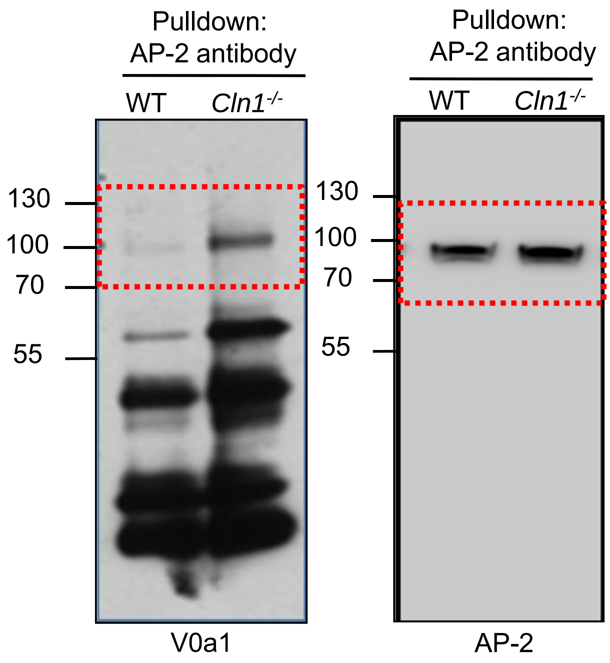

Figure 2b

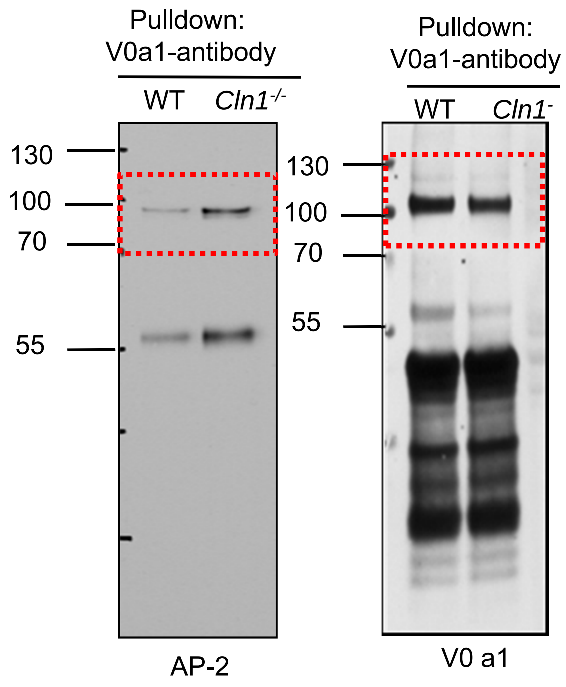

Figure 2d

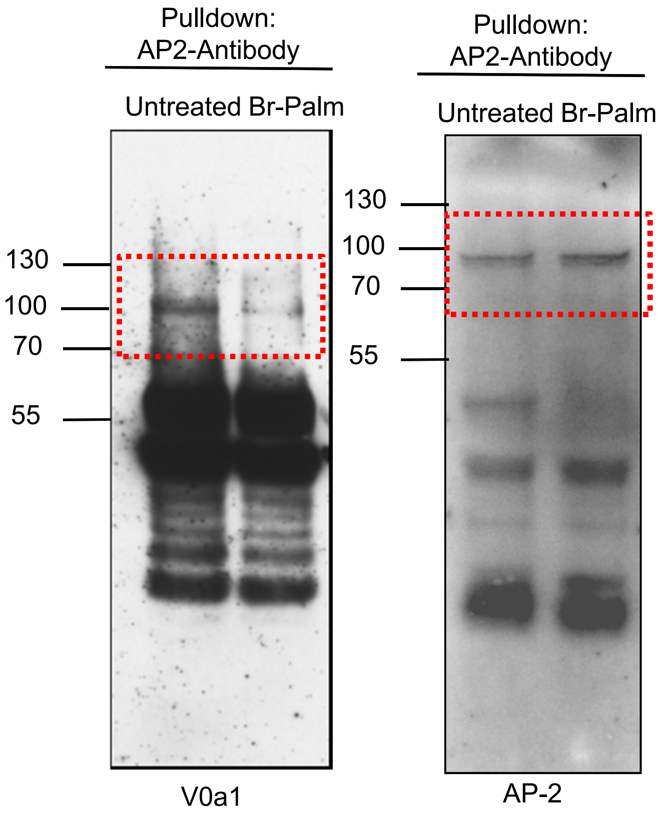

Figure 2e

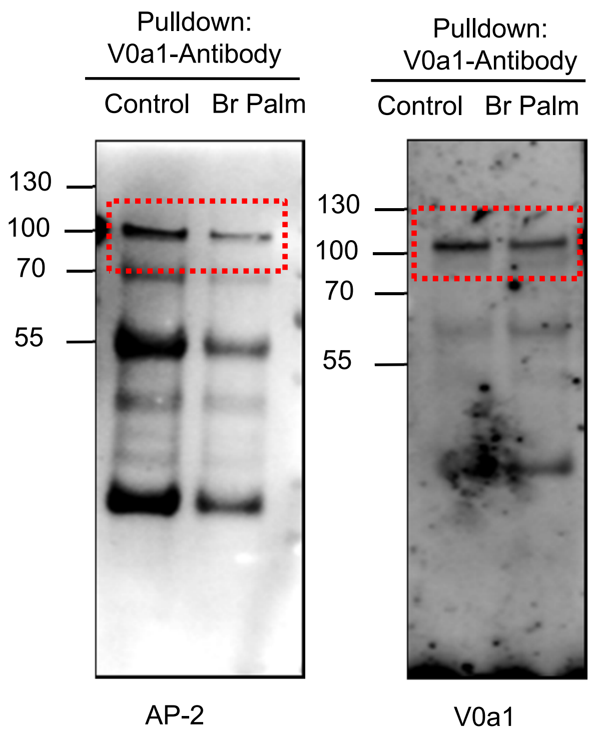

Figure 2f

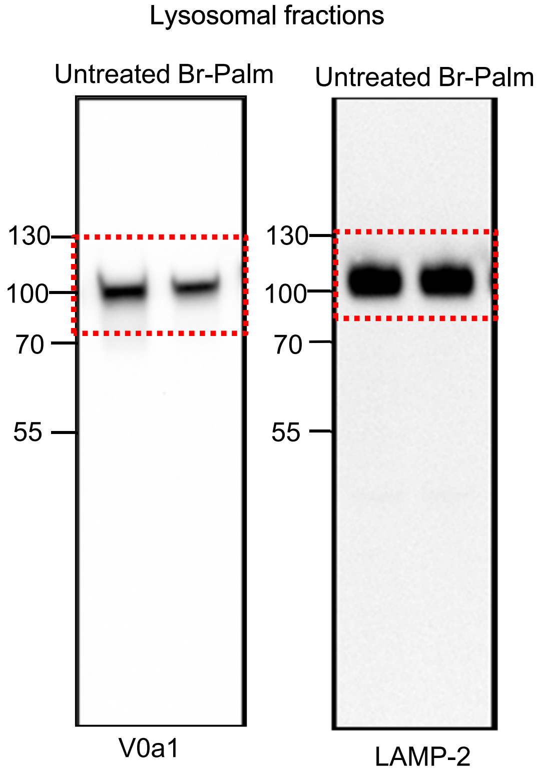

Figure 2g

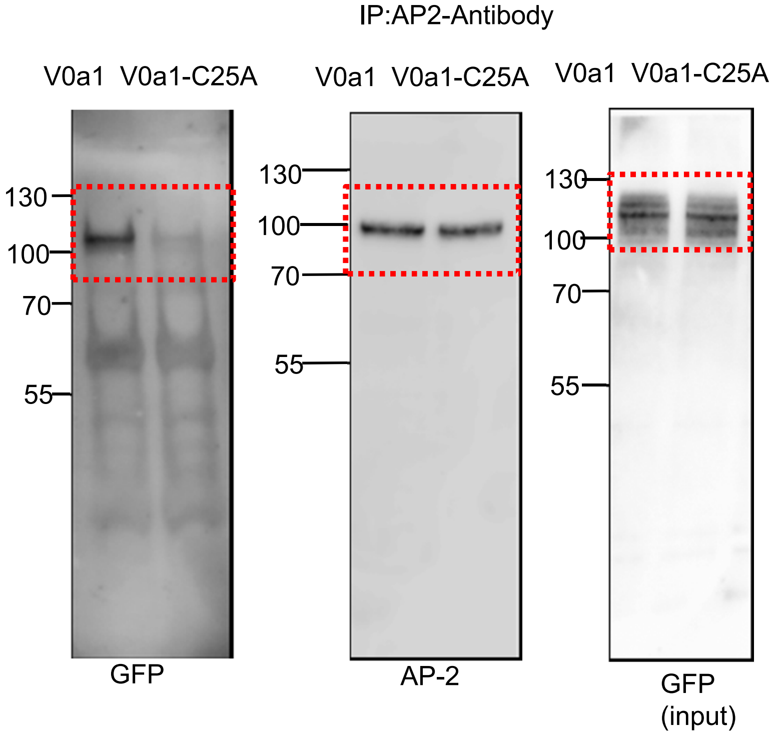

Figure 3a

Plasma membrane

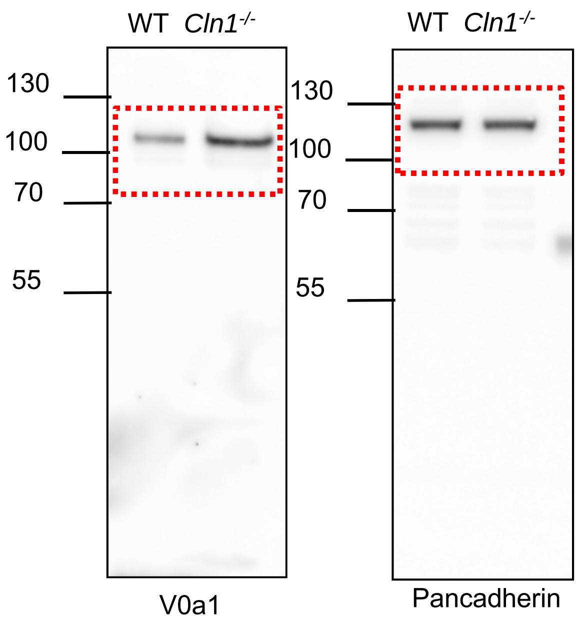

Figure 3c

Pulldown assay: AP3- δ Antibody

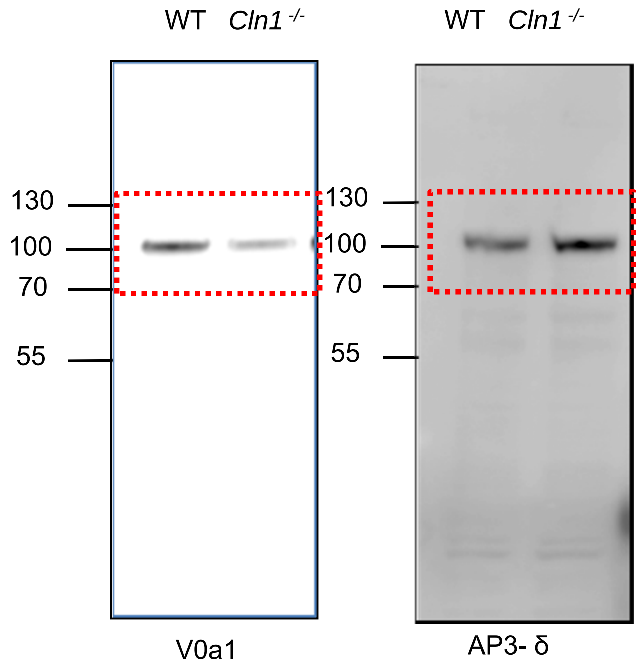

Figure 3e

Pulldown: AP3-Antibody

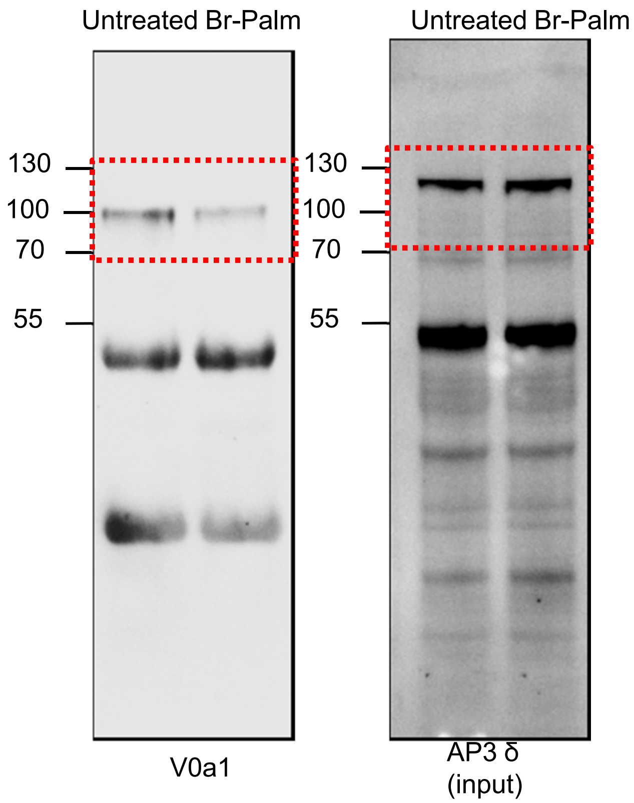

Figure 3f

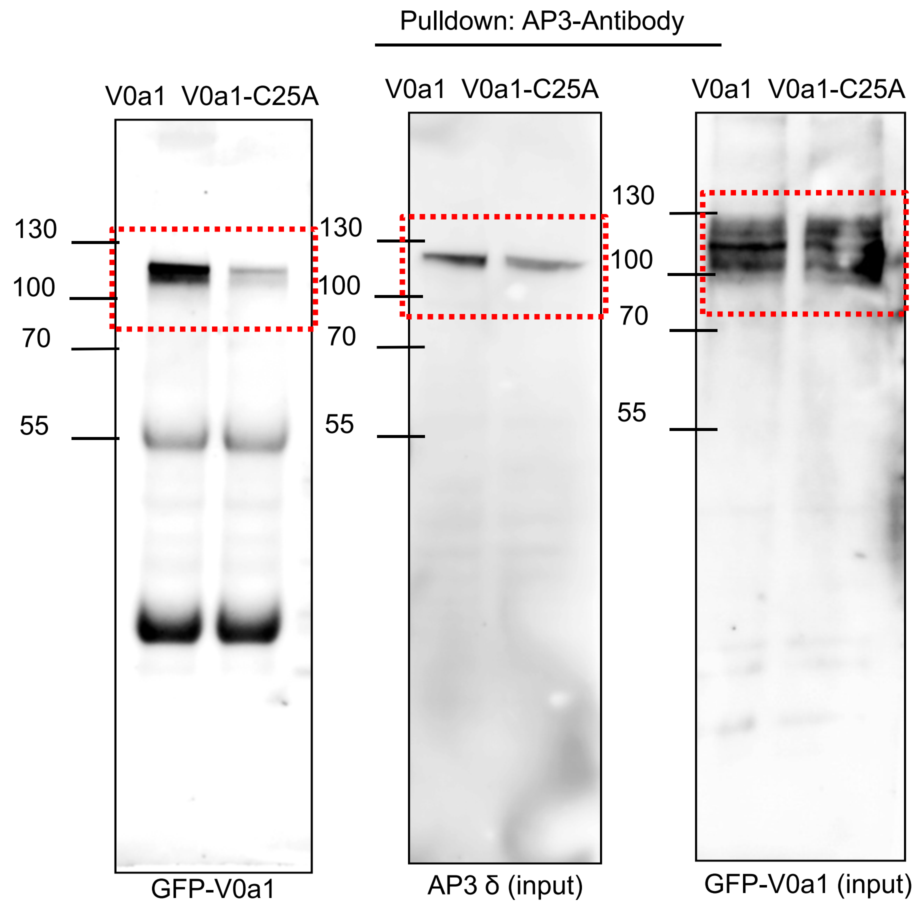

Figure 7d

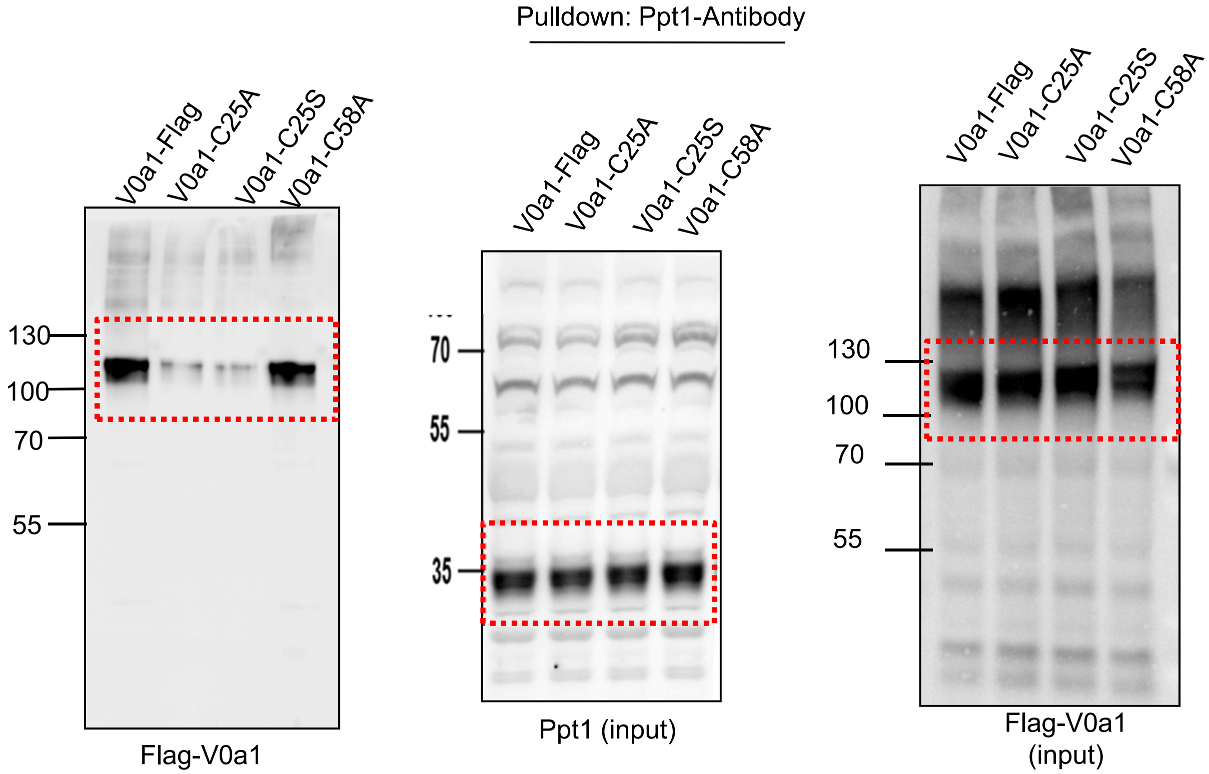

Figure 8a

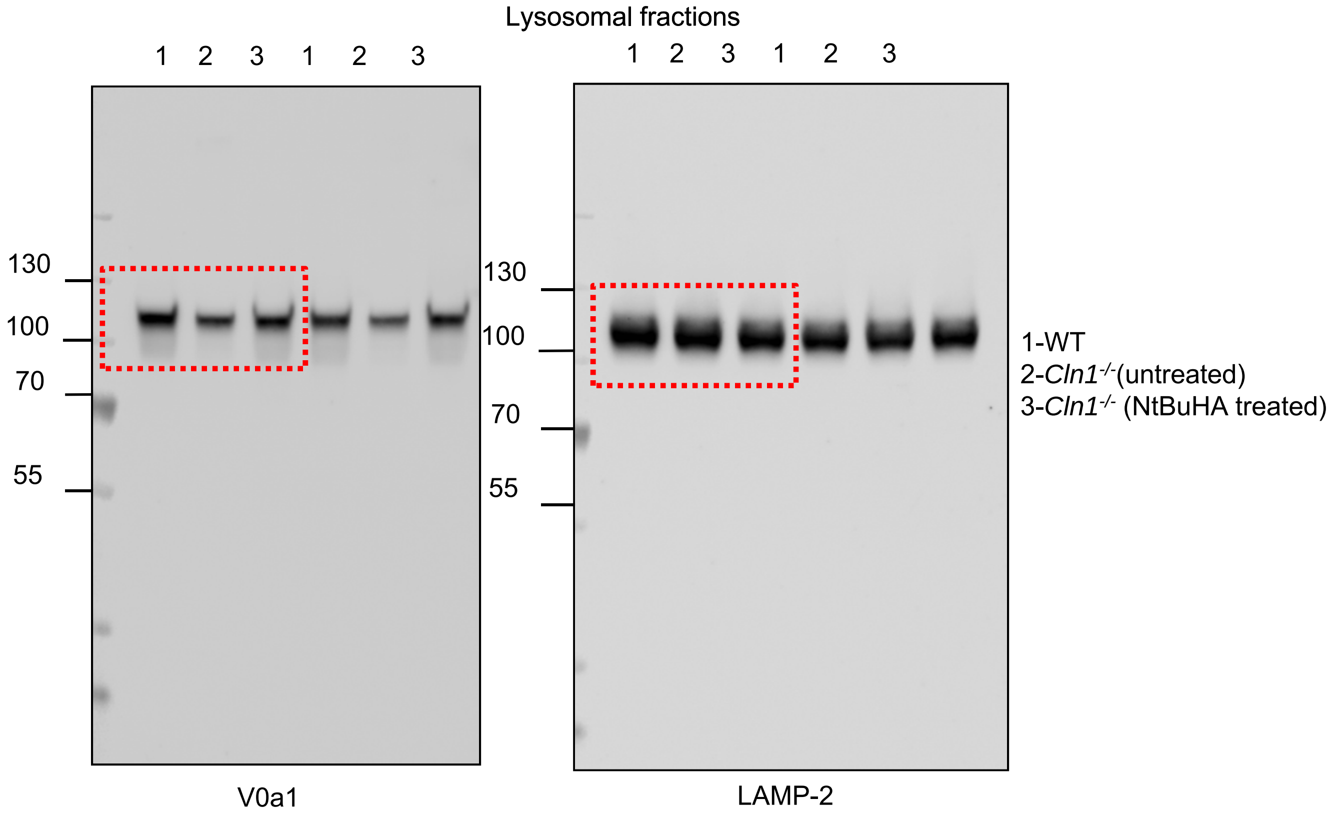

Supplementary Figure 1e

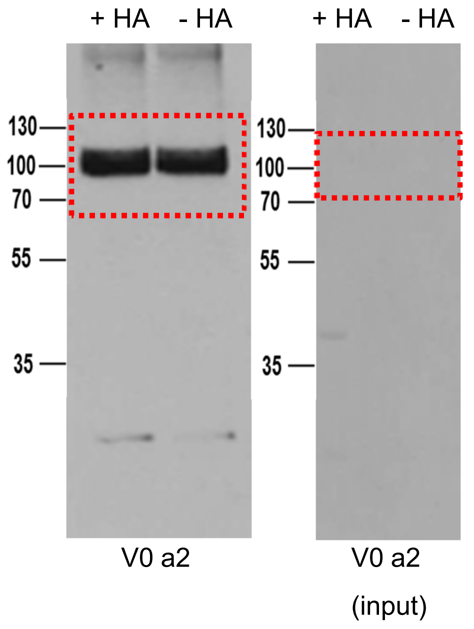

Supplementary Figure 2b

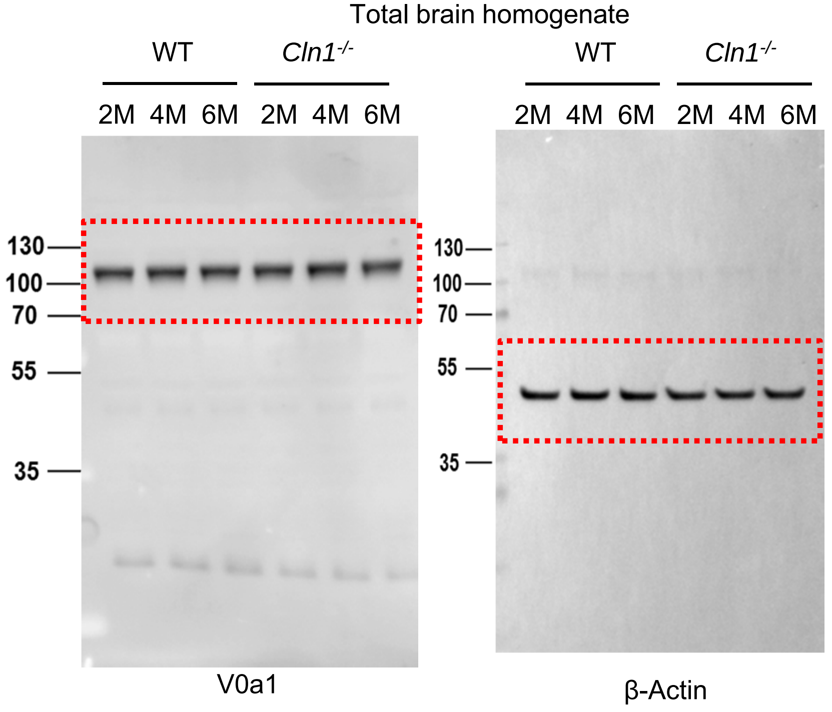

Supplementary Figure 3a

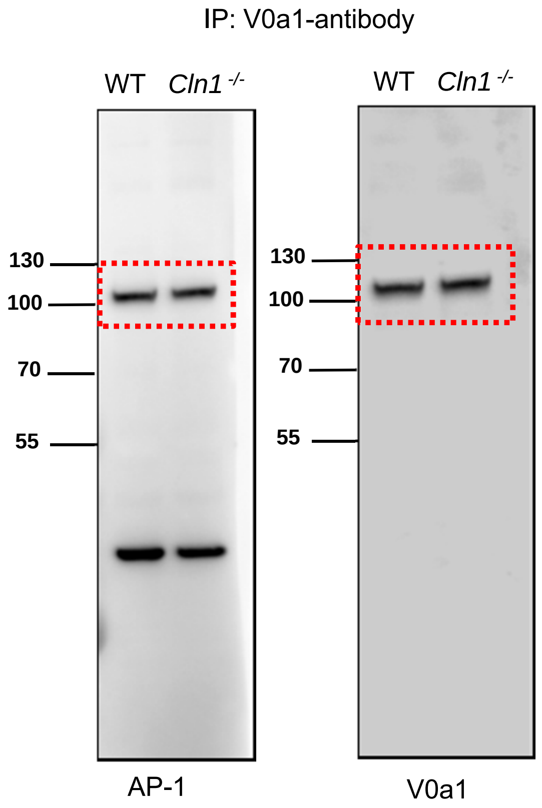

Supplementary Figure 3b

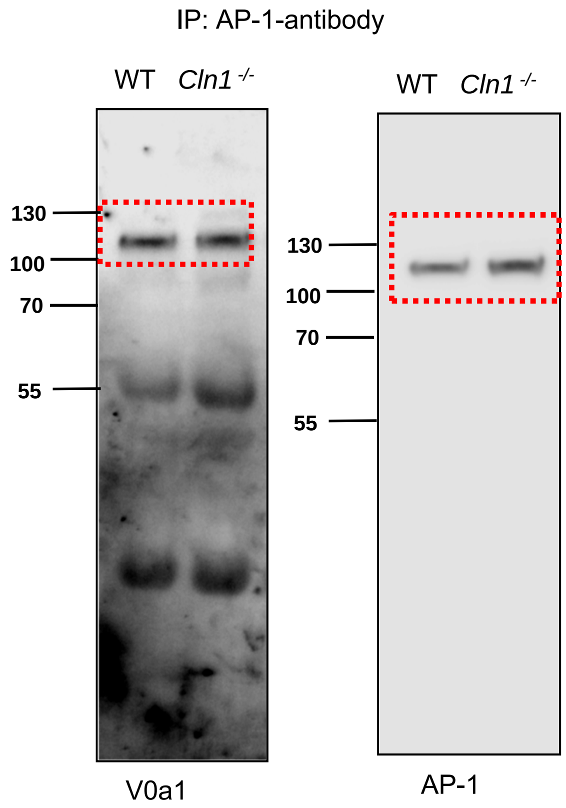

Supplementary Figure 3c

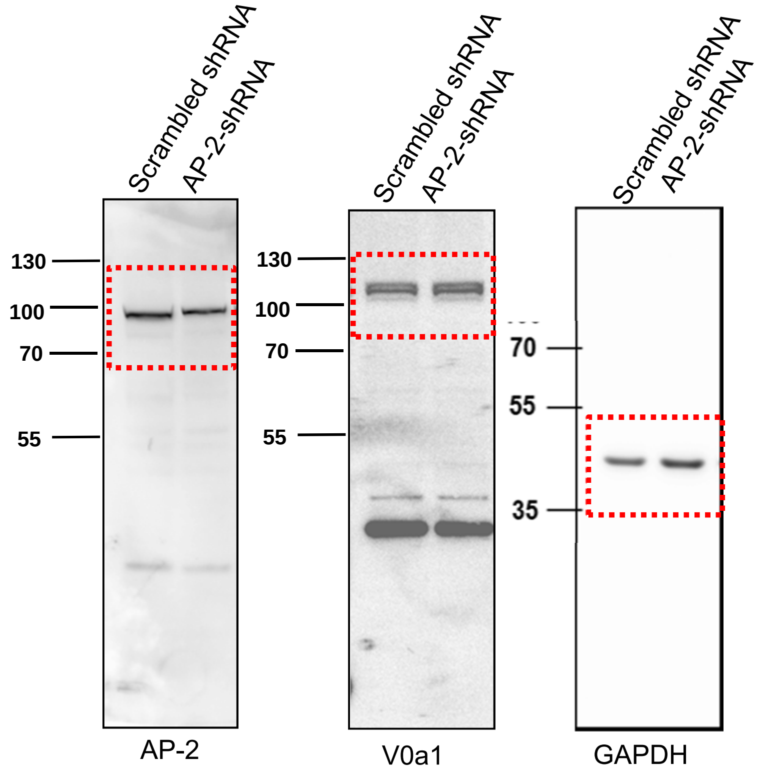

Supplementary Figure 3d

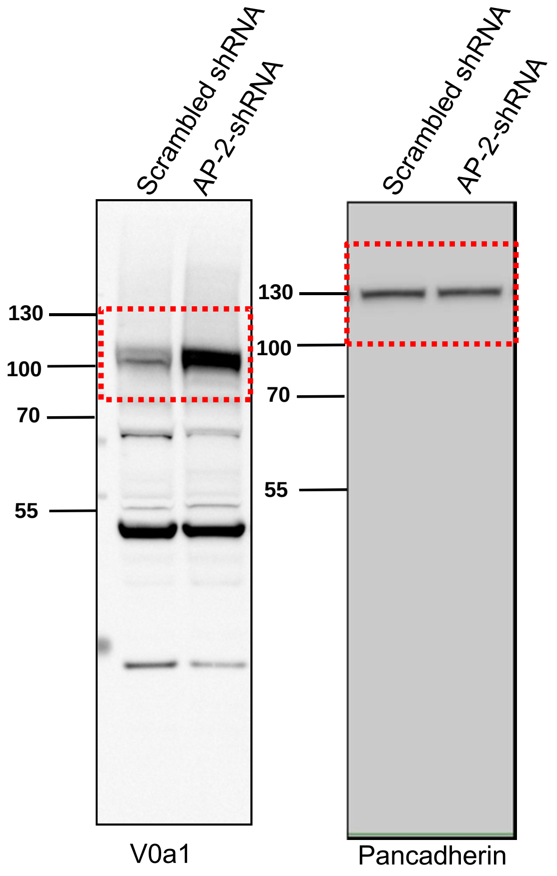

Supplementary Figure 3e

Pulldown: AP-2-Antibody

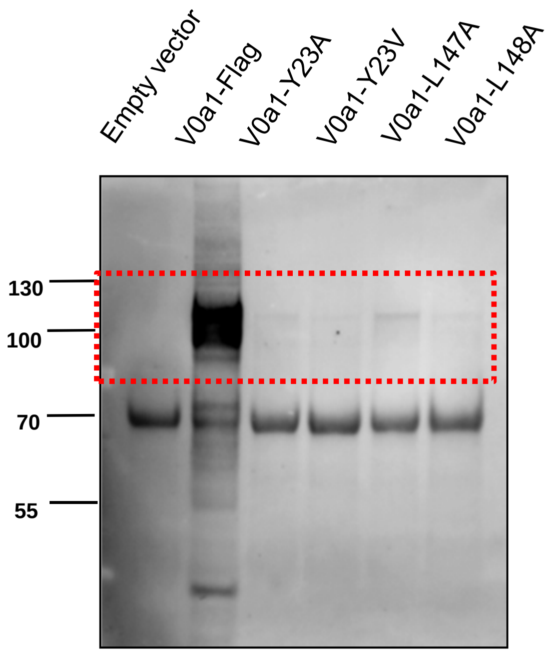

Flag V0a1

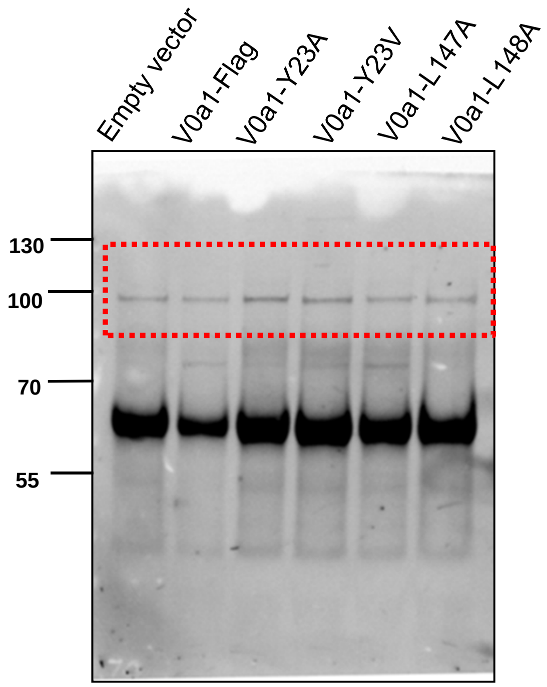

AP-2
(input)

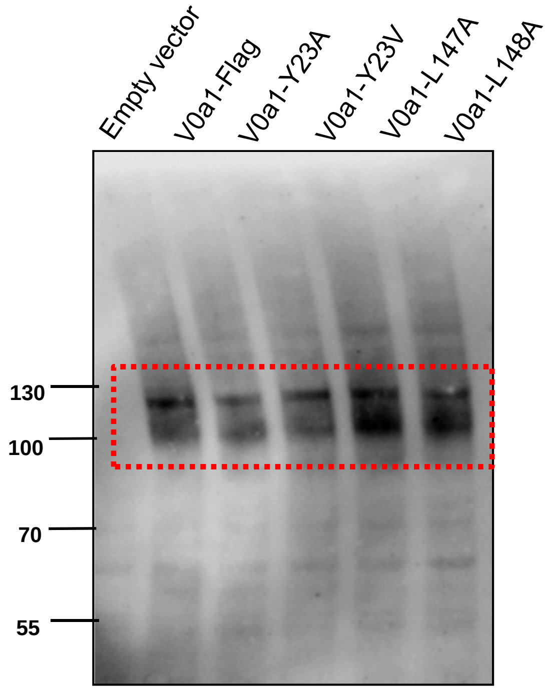

Flag V0a1
(input)

Supplementary Figure 4a

Total brain homogenate

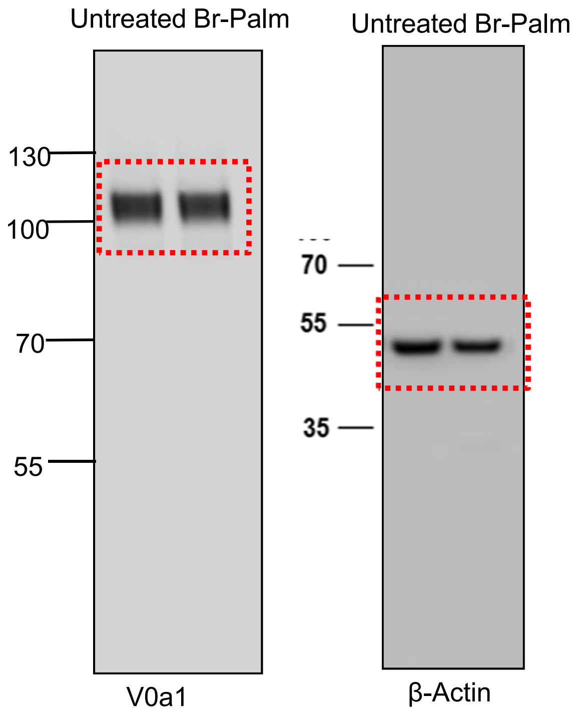

Supplementary Figure 4c

Total cell homogenate

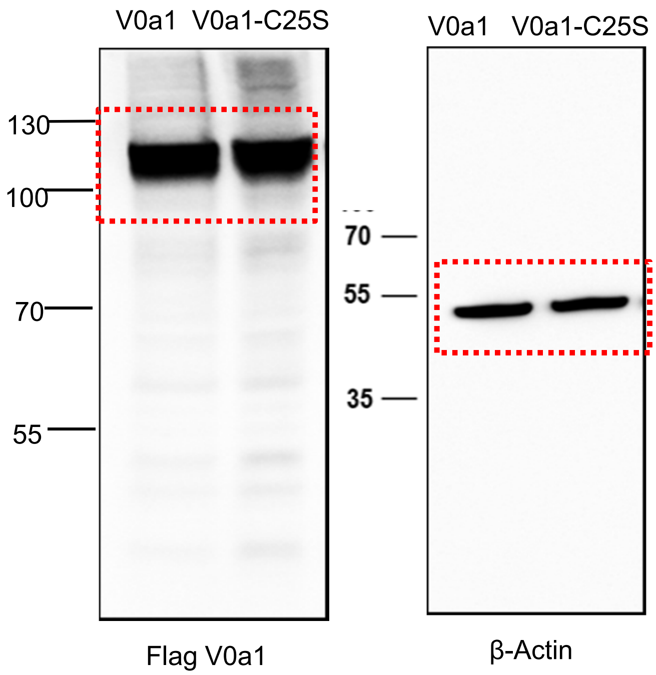

Supplementary Figure 4d

Lysosomal fractions

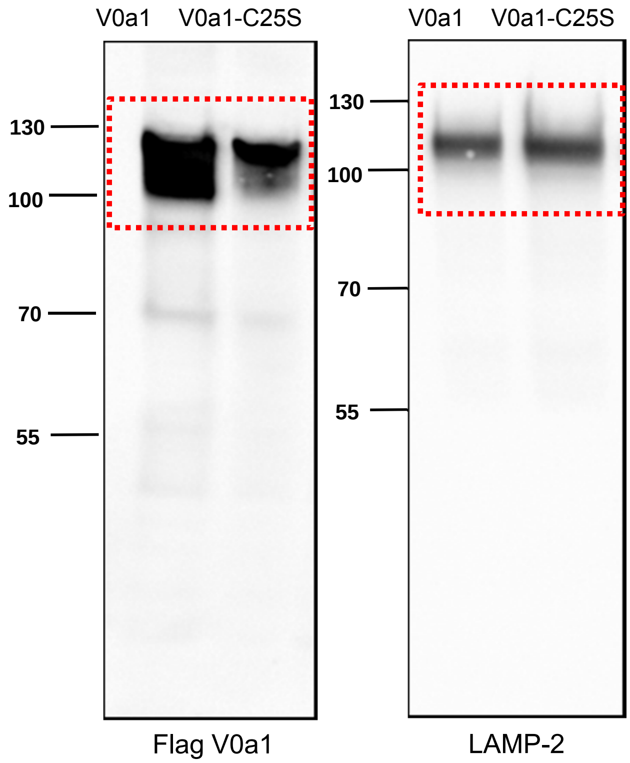

Supplementary Figure 5b

Lysosomal fraction

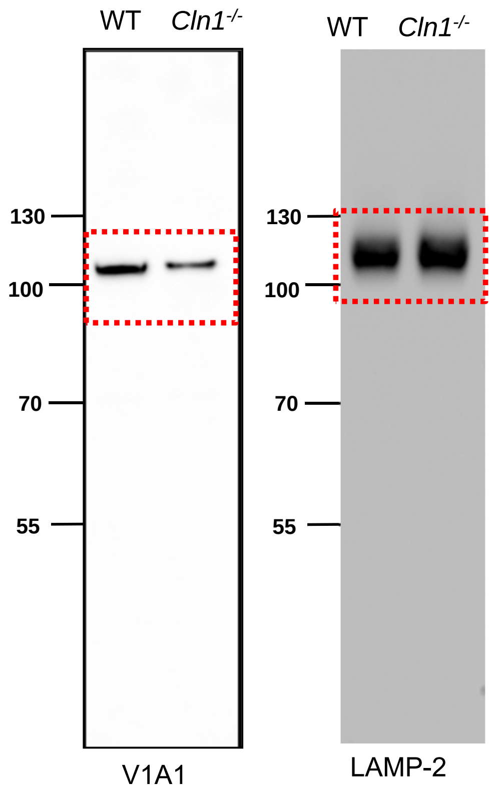

Supplement: Supplementary Information — Supplementary Figures. [file ncomms14612-s1.pdf]
